# An inhibitory gate for state transition in cortex

**DOI:** 10.7554/eLife.26177

**Published:** 2017-05-16

**Authors:** Stefano Zucca, Giulia D’Urso, Valentina Pasquale, Dania Vecchia, Giuseppe Pica, Serena Bovetti, Claudio Moretti, Stefano Varani, Manuel Molano-Mazón, Michela Chiappalone, Stefano Panzeri, Tommaso Fellin

**Affiliations:** 1 Optical Approaches to Brain Function Laboratory, Department of Neuroscience and Brain Technologies, Istituto Italiano di Tecnologia, Genova, Italy; 2 Neural Coding Laboratory, Istituto Italiano di Tecnologia, Genova and Rovereto, Italy; 3 Department of Neuroscience and Brain Technologies, Istituto Italiano di Tecnologia, Genova, Italy; 4 Neural Computation Laboratory, Center for Neuroscience and Cognitive Systems @UniTn, Istituto Italiano di Tecnologia, Rovereto, Italy; Stanford University School of Medicine, United States

**Keywords:** Neocortex, parvalbumin positive interneuron, somatostatin positive interneuron, up and down states, Mouse

## Abstract

Large scale transitions between active (up) and silent (down) states during quiet wakefulness or NREM sleep regulate fundamental cortical functions and are known to involve both excitatory and inhibitory cells. However, if and how inhibition regulates these activity transitions is unclear. Using fluorescence-targeted electrophysiological recording and cell-specific optogenetic manipulation in both anesthetized and non-anesthetized mice, we found that two major classes of interneurons, the parvalbumin and the somatostatin positive cells, tightly control both up-to-down and down-to-up state transitions. Inhibitory regulation of state transition was observed under both natural and optogenetically-evoked conditions. Moreover, perturbative optogenetic experiments revealed that the inhibitory control of state transition was interneuron-type specific. Finally, local manipulation of small ensembles of interneurons affected cortical populations millimetres away from the modulated region. Together, these results demonstrate that inhibition potently gates transitions between cortical activity states, and reveal the cellular mechanisms by which local inhibitory microcircuits regulate state transitions at the mesoscale.

**DOI:** http://dx.doi.org/10.7554/eLife.26177.001

## Introduction

The mammalian brain generates internal activities independent of environmental stimuli (Destexhe, 2011). For example, during quiet wakefulness or NREM sleep the cortex and other brain regions (e.g., the thalamus) display rhythmic electrical signals characterized by large-amplitude and low frequency (<1 Hz) oscillations (Metherate et al., 1992; Steriade et al., 1993a; Crunelli and Hughes, 2010; Halassa et al., 2014; Slézia et al., 2011; Fogerson and Huguenard, 2016; Herrera et al., 2016; Sellers et al., 2015). These peculiar activities, named slow oscillations, are dominated by recurring transitions between active (up) and silent (down) periods. In the cortex, up states are associated with sustained membrane potential depolarization in single pyramidal neurons and enhanced firing at the network level, while down states are characterized by membrane hyperpolarization and reduced circuit firing (Steriade et al., 1993b; Contreras and Steriade, 1995; Vyazovskiy et al., 2009). The alternation between these two activity states plays fundamental roles in regulating crucial cortical processes, such as the modulation of sensory inputs (Petersen et al., 2003a; Crochet et al., 2005; Haider et al., 2007; Reig and Sanchez-Vives, 2007; Reig et al., 2015), the consolidation and potentiation of memory (Marshall et al., 2006; Rasch et al., 2007), the improvement of task performance (Huber et al., 2004) and the control of synaptic plasticity (Vyazovskiy et al., 2009, 2008). Although the cellular mechanisms regulating up and down state transitions have been the focus of intense research, the role of many prominent cortical circuits in these phenomena still remains elusive. One such example is cortical inhibition (Crunelli et al., 2015). Previous studies found that inhibitory cells are active (Steriade et al., 2001; Gentet et al., 2010; Tahvildari et al., 2012; Neske et al., 2015) and that there is a tight interplay of excitatory and inhibitory conductances during the up state (Shu et al., 2003; Haider et al., 2006). Since neither a gradual buildup nor a sudden increase in inhibition near the termination of the up state was observed, these seminal data were interpreted against an active role of inhibition in the transition from an up to a down state (Shu et al., 2003; Haider et al., 2006; Neske, 2015; Sanchez-Vives et al., 2010). Moreover, in silico model of cortical dynamics showed that up-to-down transitions can occur in the presence of an activity-dependent K^+^ conductance with minor contribution of synaptic inhibition (Compte et al., 2003), further arguing against a major role of inhibition in shaping network shifts from the up to the down state. However, pharmacological blockade of GABA_A_ (Sanchez-Vives et al., 2010) and GABA_B_ (Mann et al., 2009) receptors significantly modifies the duration and the frequency of up and down states and other modelling work showed that inhibition may actually contribute to facilitate the up-to-down transition (Bazhenov et al., 2002; Chen et al., 2012). Furthermore, paired recordings in anesthetized and naturally sleeping cats demonstrated that bursts of inhibitory activity do precede the onset of the down state, suggesting a potential role of inhibition in the control of up-to-down transitions (Lemieux et al., 2015).

In this study, we combined two-photon targeted single neuron electrophysiological recordings, phase-locking analysis, and cell-specific optogenetic perturbations in anesthetized and non-anesthetized awake mice to investigate the *causal* contribution of specific inhibitory circuits, including parvalbumin (PV) and somatostatin (SST) interneurons, in the regulation of cortical state transitions. Our data demonstrate that optogenetic modulation of interneurons precisely regulates network up-to-down state shifts and reveals a previously unacknowledged role of the inhibitory network in the control of down-to-up state transitions. This bidirectional (*up-to-down* and *down-to-up*) inhibitory control of state transition is interneuron-type specific and finely controlled at the microcircuit level, with local ensemble of active interneurons gating state transitions over millimetres of cortical territories.

## Results

**Figure 1.**
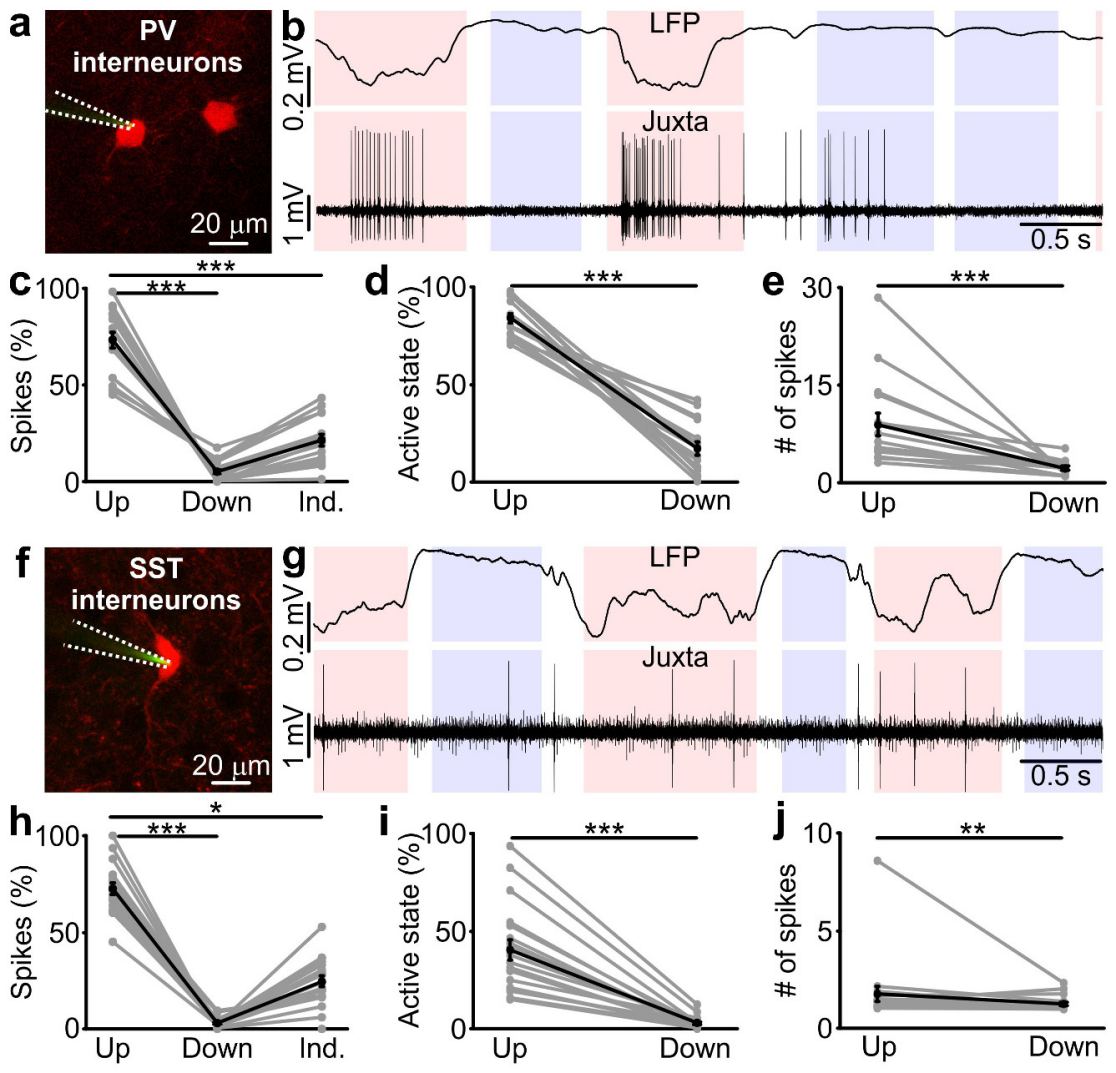
Firing activity of PV and SST interneurons during cortical up and down states in vivo. (**a**) Fluorescence image showing a glass pipette (dotted white line) used for juxtasomal recordings from a PV positive interneuron (red cell) in a PV*x*TdTomato bigenic transgenic animal. (**b**) Representative traces of simultaneous LFP (top) and juxtasomal (bottom) recordings from an identified PV interneuron during up and down states. Pink and purple colours in the background indicate up and down states that were identified from the LFP signal, respectively. The white background colour indicates indeterminate states (see Materials and methods). (**c**) Action potentials fired by PV cells in the three identified periods (up, down and indeterminate, p = 7E-8, one-way ANOVA, N = 16 cells from five animals). In this as well in other figures: grey dots and lines indicate single experiment; black dots and lines indicate the average value represented as mean ± s.e.m; n.s., p>0.05; *p<0.05; **p<0.01; ***p<0.001. (**d**) Percentage of active up states and active down states (p = 3E-16, unpaired Student’s *t*-test, N = 16 cells from five animals). (**e**) Number of spikes fired by PV cells per single up or down state (p = 8E-6, Mann-Whitney test, N = 16 cells from five animals). (**f–g**) Same as in a, b but in SST*x*TdTomato bigenic transgenic animals. (**h–j**) Same as in c-e for SST interneurons. In h, p = 2E-8, Friedman test, N = 19 cells from seven animals. In i, p = 1E-7, Mann-Whitney test, N = 19 cells from seven animals. In j, p = 9E-3, Mann-Whitney test, N = 19 cells from seven animals. **DOI:** http://dx.doi.org/10.7554/eLife.26177.002 10.7554/eLife.26177.003Figure 1—source data 1.Source data for the analysis of the firing activity of PV and SST interneurons during up and down states.**DOI:** http://dx.doi.org/10.7554/eLife.26177.003

To determine if and how PV and SST interneurons control state transitions, we first characterized the spiking activities of these two cellular subpopulations during spontaneous up and down states in anesthetized mice. To this aim, we performed two-photon-targeted juxtasomal electrophysiological recordings in *Pvalb^Cre^* (here called PV-Cre) *x* TdTomato and *Sst^Cre^* (here called SST-Cre) *x* TdTomato mice while simultaneously monitoring network activities with a LFP electrode (Figure 1).

**Figure 1—figure supplement 1.**
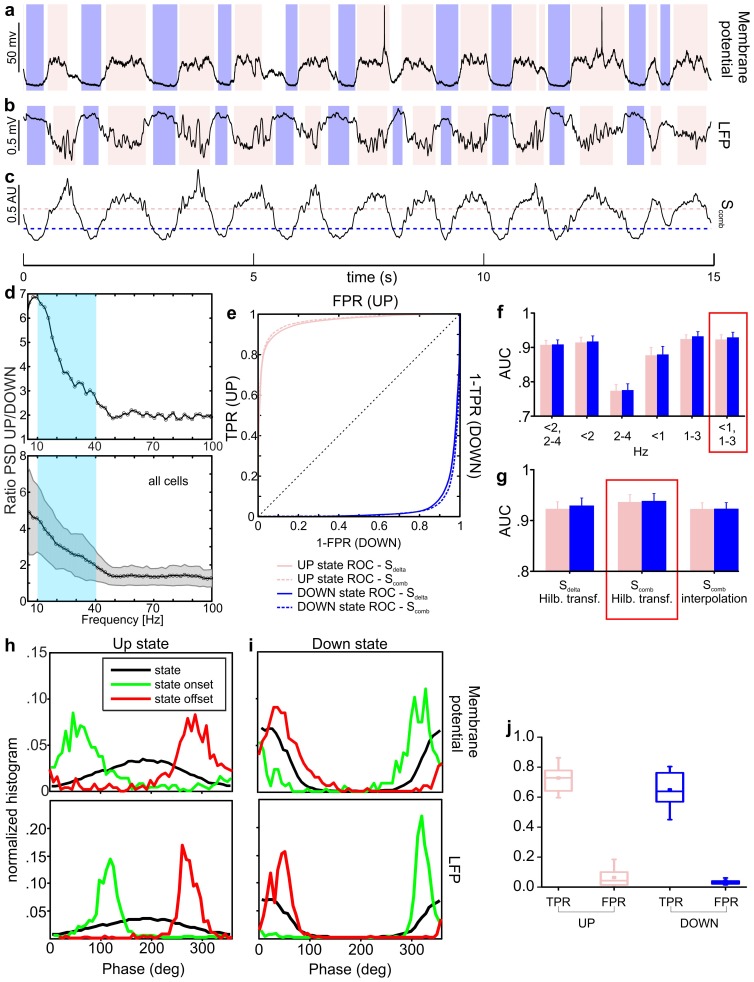
Up and down state detection from the LFP signal. (**a–c**) Representative example of up/down state detection results. Up/down state periods (pink and purple shadows, respectively) as detected from membrane potential are shown in a, whereas states detected in the LFP signal (and based on the decision variable trace shown in c) are reported in b. In c, the up state threshold is marked by the dotted pink line, whereas the down state threshold by the purple dotted line. (**d**) Ratio between power spectral densities (PSD) of LFP in up/down states (representative cell in upper panel, mean ± s.d. of all cells in lower panel). The light blue square indicates the 10–40 Hz range used in our algorithm. (**e–g**) ROC curves for the detection of up (pink) and down (purple) states using either S_delta_ or S_comb_ are reported in e (representative cell). Up/down state ROC curves are shown either in the top left or in the bottom right half of the xy plane, respectively. The area under ROC curve (AUC) using different frequency bands for the computation of S_delta_ is shown in f. The AUC for the various metrics (i.e. S_delta_ or S_comb_) or phase computation methods (i.e. Hilbert transform or interpolation) are shown in g. Red rectangles highlight the combinations of parameters giving the maximum performance. (**h–i**) Statistical distributions of instantaneous phase during up/down states (up in h, down in i, black curves), state onset (in green), and state offsets (in red). Upper panels refer to state detection based on membrane potential, lower panels to state detection based on LFP. (**j**) True positive (TPR) and false positive rates (FPR) obtained by detecting up/down states on S_comb_ traces ([0, 1]-[1, 3] Hz frequency bands, instantaneous phase computed by Hilbert transform, and thresholds set as described in Materials and methods). **DOI:** http://dx.doi.org/10.7554/eLife.26177.004 10.7554/eLife.26177.005Figure 1—figure supplement 1—source data 1.Source data for Up and Down state detection from the LFP signal.**DOI:** http://dx.doi.org/10.7554/eLife.26177.005

Up states (pink in Figure 1b,g) and down states (purple) were identified from the LFP using an established method (Saleem et al., 2010; Mukovski et al., 2007) which was fine-tuned to our experiment by validating it against ‘ground truth’ data made of simultaneous recordings of membrane potentials of pyramidal neurons and the LFP (see Materials and methods and Figure 1—figure supplement 1). Importantly, this method optimally combined several variables extracted from the LFP to best estimate the network state (up or down, Figure 1—figure supplement 1). A crucial variable in this detection algorithm was the LFP phase in the low frequency band, which could thus be considered a good indicator of network state (Saleem et al., 2010) (see also Materials and methods). Phases between 112 and 264 degrees (the ‘trough’ regions of the LFP slow oscillatory component) mainly corresponded to up states and phases between 322 and 45 degrees (the ‘peak’ LFP regions) mainly corresponded to down states (Figure 1—figure supplement 1h,i). The LFP-based identification of states performed well on ground truth data, minimizing the percentage of misclassified periods (i.e., false positives) at 6.0 ± 0.2% and 4.0 ± 0.1% for up and down states, respectively (see Materials and methods and Figure 1—figure supplement 1j).

Both interneuron types preferentially discharged action potentials (APs) during up states (Figure 1c,h) in agreement with previous reports (Puig et al., 2008; Tahvildari et al., 2012; Neske et al., 2015). However, we found that a small but significant fraction of spikes (p<1E-2 in 16 out of 16 PV cells from five animals and p<4E-2 in 15 out of 19 SST neurons from seven animals, see Materials and methods for details on the statistical test) occurred during down states both for PV and SST cells (Figure 1c,h). The percentage of up states in which the recorded interneuron fired (named active up states) was higher than the percentage of down states displaying cell firing (called active down states) (Figure 1d,i) and the number of spikes per active up state was higher than the number of spikes per active down state for both PV cells and SST interneurons (Figure 1e,j). Moreover, the average firing rate, the percentage of active up and active down states, and the average number of spikes fired during active up or down states was significantly higher for PV cells compared to SST interneurons (active up states, p=4E-8, unpaired Student’s *t*-test; active down states, p=2E-4, Mann-Whitney test; # of spikes per active up state, p=3E-6, Mann-Whitney test; # of spikes per active down state: p=1E-3, Mann-Whitney test; PV, N = 16 cells from five animals; SST, N = 19 cells from seven animals).

### Interneuron firing correlates with changes in the low frequency LFP phase

**Figure 2.**
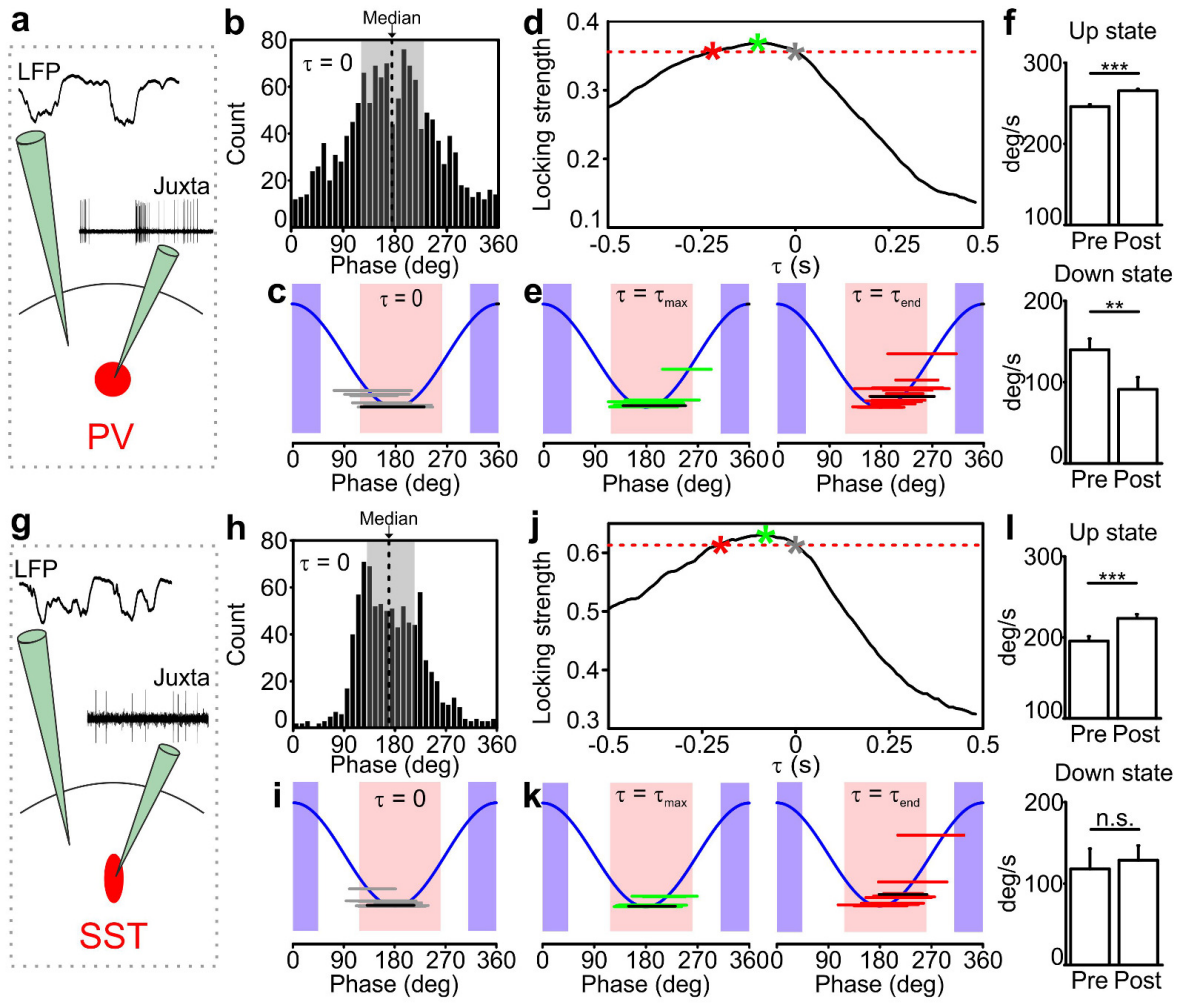
Temporal correlation between the activity of PV and SST interneurons and the LFP. (**a**) Schematic representation of the experimental configuration for simultaneous LFP recording and fluorescence targeted juxtasomal recordings from PV interneurons. (**b**) Phase of firing distribution of one representative PV interneuron in the absence of temporal shift (τ = 0). The dashed line indicates the median. The shaded area indicates the range of preferred phase of firing defined as between the 25th and the 75th percentile. (**c**) The horizontal grey lines indicate the range of preferred phase of firing for each recorded cell. The blue lines plot a cosine function used to show the phase convention in terms of LFP peaks and troughs. The crossing between each grey line and the blue sinusoid occurs at the median value of phase of firing for each recorded cell. The black line represents the values of the cell shown in b. Pink and purple regions indicate phase ranges belonging to up states and down states, respectively. (**d**) Locking strength as a function of the time shift τ for the representative PV interneuron displayed in b. The grey asterisk indicates the locking strength corresponding to the τ = 0 value, the green asterisk that corresponding to the τ_max_ value and the red asterisk that corresponding to the τ_end_ value. (**e**) Same as in c but in presence of temporal shift τ = τ_max_ (left panel) and τ = τ_end_ (right panel). (**f**) LFP phase speed averaged over 200 ms before any PV interneuron spike lying between −400 ms and −200 ms from a state end and the LFP phase speed averaged over 200 ms after the same spikes. The post-spike phase speed is significantly higher than the pre-spike phase speed in up states (top panel), while it is significantly smaller in down states (bottom panel, for up states, p=2E-13, one-tailed paired Student’s *t*-test, N = 8911 stretches from 16 cells; for down state, p=8E-3, one-tailed paired Student’s *t*-test, N = 199 stretches from 11 cells). (**g–k**) Same as in a-e but for SST interneurons. (**l**) Spike-triggered phase speed analysis of SST interneurons as in f: the post-spike phase speed is significantly larger than the pre-spike phase speed in up states (top panel, p=2E-5, one-tailed paired Student’s *t*-test, N = 1346 stretches from 15 cells), while there is no significant difference between the post-spike and the pre-spike phase speed in down states (bottom panel, p=0.6, one-tailed paired Student’s *t*-test, N = 12 stretches from 7 cells). **DOI:** http://dx.doi.org/10.7554/eLife.26177.006 10.7554/eLife.26177.007Figure 2—source data 1.Source data for the analysis of the preferred phase of firing for PV and SST interneurons.**DOI:** http://dx.doi.org/10.7554/eLife.26177.007 10.7554/eLife.26177.008Figure 2—source data 2.Source data for the analysis of the spike triggered phase speed velocity.**DOI:** http://dx.doi.org/10.7554/eLife.26177.008

The results in Figure 1 suggest that PV and SST interneurons do not fire uniformly during the LFP slow oscillation cycle, for example with more elevated firing during the up state with respect to down state. To quantitatively investigate this temporal relationship, we computed, for each recorded neuron, the distribution of phase at the exact time at which spikes were fired (i.e. ‘phase of firing’ distribution, Figure 2). This is shown in Figure 2b,h for one representative PV cell and one representative SST neuron, respectively. Locking to the slow wave phase was significant for all 16/16 PV and 15/15 SST neurons (Rayleigh test, p<2E-30 for PV interneurons and p<7E-7 for SST interneurons, see also Materials and methods). Across the population PV and SST neurons preferentially fired during the first half of the up state and the average preferred phase of firing was 167 ± 5 degrees and 170 ± 5 degrees for PV and SST interneurons, respectively (Figure 2c,i). However, these neurons fired also during phases associated to the second half of the up state, as exemplified by the spread of the phase of firing distribution over phase angles in Figure 2c,i. The phase bins characterized by strong firing were also those where spikes were fired more reliably across trials (Figure 2—figure supplements 1 and 2, see also Materials and methods).

**Figure 2—figure supplement 1.**
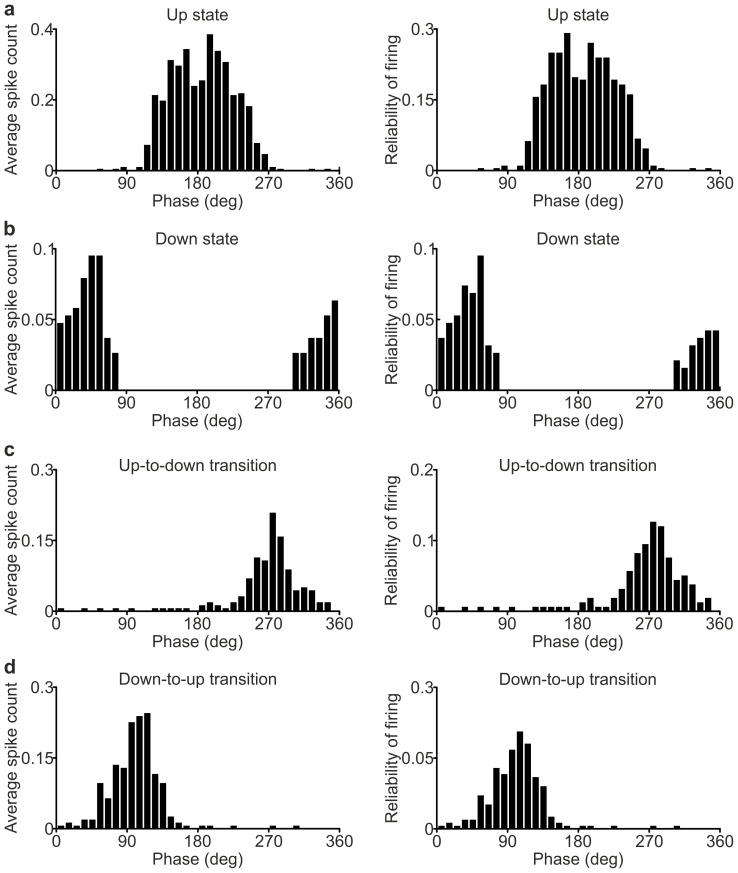
Phase of firing strength and phase of firing reliability in PV interneurons. (**a**) Histogram of the phase of firing strength (left) and histogram of the phase of firing reliability (right) for the representative PV interneuron showed in Figure 2b. Only spikes occurring during up states were considered. (**b–d**) Same as in a for spikes fired during down states (**b**), up-to-down transitions (**c**) and down-to-up transitions (**d**). **DOI:** http://dx.doi.org/10.7554/eLife.26177.009

**Figure 2—figure supplement 2.**
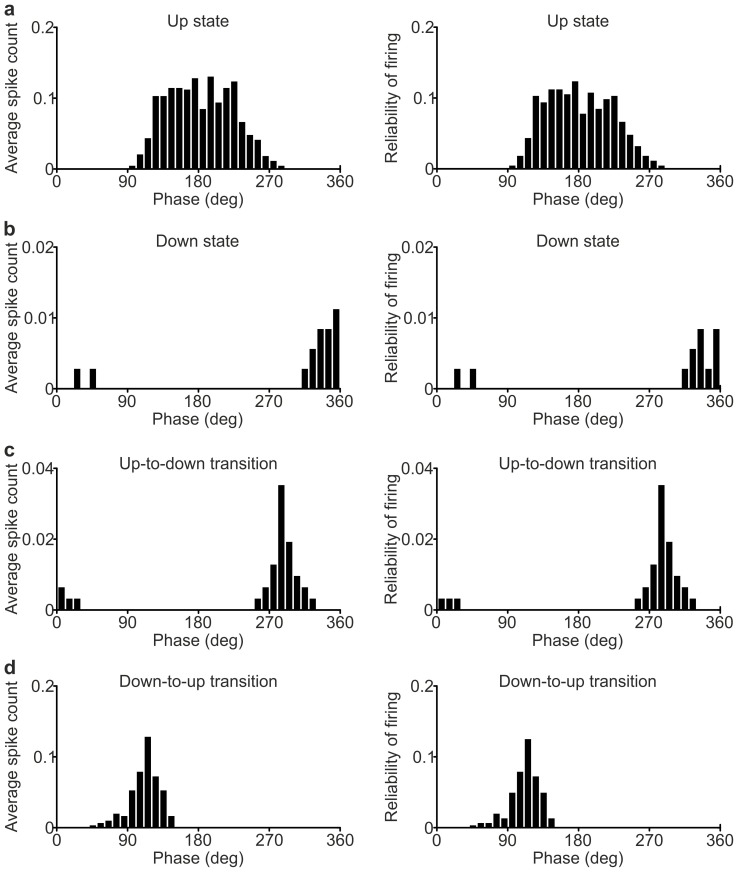
Phase of firing strength and phase of firing reliability in SST interneuron*s*. (**a**) Histogram of the phase of firing strength (left) and histogram of the phase of firing reliability (right) for the representative SST interneuron showed in Figure 2h. Only spikes occurring during up states were considered. (**b–d**) Same as in a for spikes fired during down states (**b**), up-to-down transitions (**c**) and down-to-up transitions (**d**). **DOI:** http://dx.doi.org/10.7554/eLife.26177.010

**Figure 2—figure supplement 3.**
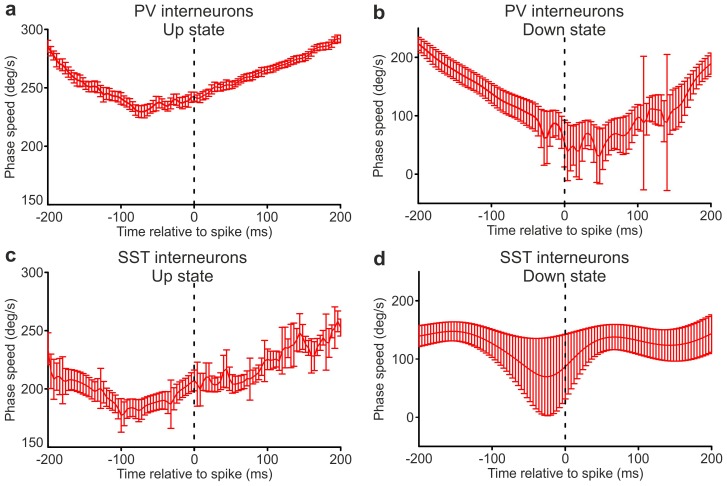
Spike-triggered phase speed across the times of interneuron spikes close to state end. (**a**) Phase speed of the recorded LFP (mean ± s.e.m.) triggered on a PV interneuron spike that was fired between 400 and 200 ms before the end of an up state, as a function of time. The spikes were selected with the only criterion that the entire stretch of considered phase speed lays within the same up state. (**b**) Same as in a, but the phase speed corresponds to down states. (**c–d**) as in a-b, but the analysed phase speed was triggered on SST interneuron spikes. **DOI:** http://dx.doi.org/10.7554/eLife.26177.011

**Figure 2—figure supplement 4.**
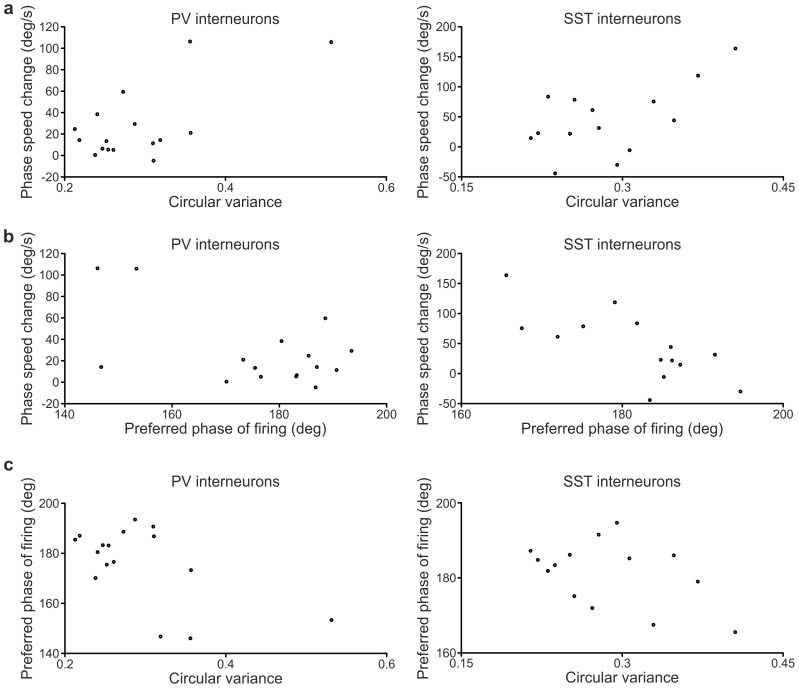
Correlation between LFP phase speed changes and firing properties of cortical interneurons. (**a**) Plots of the LFP phase speed changes triggered by spikes close to the end of up states as a function of the circular variance of the phases of firing distributions in up states for PV (left) and SST (right) interneurons. Each dot represents one recorded cell. In this as well as in the other panels, only cells with at least ten data stretches (see Materials and methods) in the up state were considered. (**b**) LFP phase speed changes triggered by spikes close to the end of up states as a function of the preferred phase of firing in up states of PV (left) and SST (right) cells. (**c**) Correlation plots between the preferred phase of firing for PV (left) and SST (right) interneurons and the circular variance of the phases of firing distributions in up states. **DOI:** http://dx.doi.org/10.7554/eLife.26177.012

The relationship between spike timing of PV/SST interneurons and the phase of slow LFP oscillation can imply either that the firing of interneurons causally leads to changes in network phase (and thus causes changes in network states) or that interneurons are entrained to the slow oscillation of network activity and thus fire at specific phases. To disambiguate between these two scenarios, we followed (Siapas et al., 2005; Eschenko et al., 2012) to take advantage of the fact that the slow oscillation is not constant in frequency but shows small frequency fluctuations over time. We shifted the LFP backward or forward by an amount τ, and we computed the strength of phase locking of spikes (quantified as one minus the circular variance of the phase of firing distribution) as a function of the time shift τ for each recorded interneuron. We reasoned that if spike times have a causal effect on slow wave dynamics, then the LFP phase dynamics will be better predicted by the structure of past spike times, thereby leading to higher phase locking values for *negative* shifts of the LFP with respect to the firing activity of interneurons. In the absence of such causal effect, that is, slow network oscillations determining when interneurons fire, we expect that LFP phase dynamics better relate to the structure of spike times in the future, thereby leading to higher phase locking values for *positive* shifts of the LFP.

The dependence of the locking strength on τ is shown in Figure 2d,j for one representative PV cell and one SST neuron, respectively. Typically, the locking strength was larger for negative time shifts and it was maximal for negative time shifts τ in 15/16 PV and 13/15 SST neurons. This suggests that the spike-phase relationships reflect that firing of interneurons causes changes in phase dynamics more than they reflect interneuron’s firing being enslaved by the slow oscillatory component of the LFP. We estimated the temporal extent of the putative causation of interneurons on the slow LFP oscillation as the range of negative time shifts for which the time-shifted phase locking was significant (p<0.01, Rayleigh test, Bonferroni corrected) and higher than the maximal value of time shifted locking for τ ≥ 0 (i.e., higher than the maximal value that could be explained by non-causal effects only). The average value of the maximal temporal extent of causation, τ_end_ (red asterisk in Figure 2d,j), was −221 ± 41 ms for PV interneurons and −189 ± 30 ms for SST interneurons. The value of the maximal causation, τ_max_ (green asterisk in Figure 2d,j), was −93 ± 19 ms, significantly lower than zero (p = 1E-4, one-tailed one-sample Student’s t-test, N = 16 from five animals) for PV cells and −81 ± 13 ms (p = 1E-5, one-tailed one-sample Student’s t-test, N = 15 cells from seven animals) for SST neurons.

To better visualize what these causation time ranges may imply, we computed the preferred phase of firing as the median of the corresponding phase of firing distribution in both PV and SST interneurons for τ = 0, τ = τ_max_ and τ = τ_end_. As displayed in Figure 2c,i, when τ = 0 both PV and SST interneurons preferentially fire during the first part of the up state. For τ = τ_max_ and τ = τ_end_, the preferred phase of firing was shifted for both classes of interneurons towards the second half and the end of the up state (for τ = τ_max_, 192 ± 6 degrees and 190 ± 4 degrees for PV and SST, respectively, Figure 2e,k, left panels; for τ = τ_end_, 215 ± 7 degrees and 213 ± 9 degrees for PV and SST respectively, Figure 2e,k, right panels). This analysis suggests that the firing activity of interneurons, which primarily occurred in the first part of the up state, exerts maximal causation in the latter part of the up state, including its end.

To investigate the nature of such statistical influences of spikes on future changes in slow LFP dynamics and state ends, we further analyzed the changes in LFP phase speed in proximity to the state ends, over the putative causal time range determined above. Given that phase is a good proxy of state dynamics, decreases and increases in phase speed following a spike near the end of a state may be interpreted as the spike delaying or anticipating the state end, respectively. We quantified the mean changes in phase speed triggered to a spike time prior to the end of an up and down state (Figure 2f,l and Figure 2—figure supplement 3). We found that, on average, phase speed significantly increased after a PV spike (Figure 2f, top panel) and a SST spike (Figure 2l, top panel) near the end of an up state. In contrast, phase speed significantly decreased (Figure 2f, bottom panel) after a PV spike near the end of a down state, while no significant effect (Figure 2l, bottom panel) was found for SST neurons. This latter result is probably influenced by the paucity of recorded spikes in the considered time range (see Figure 2 legend). To rule out the possibility that these changes in phase speed are related to stereotyped asymmetries in LFP shapes near the end of a state that have nothing to do with the spiking activity of interneurons, we compared these spike-triggered phase speed results with ‘control’ samples of LFP phase speed near state ends observed in the absence of interneuron spikes (see Materials and methods for details). Importantly, we found that the decreases in speed in down states for PV cells and the increases in speed in up states for PV and SST neurons around the center of the control stretches, computed exactly as with the real data triggered to a spike time, were not significant (for up state stretches, p = 8E-1, one-tailed paired Student’s *t*-test, N = 8911 stretches from 16 PV cells and p = 9E-1, N = 1346 stretches from 15 SST cells; for down state stretches, p = 1E-1, one-tailed paired Student’s *t*-test, N = 199 stretches from 11 PV cells and p = 2E-1, N = 12 stretches from 7 SST cells). Thus, we conclude that our results could not be explained purely by stereotyped asymmetries in the LFP wave near state ends that are observed also in the absence of interneuron spikes.

To investigate whether any of the phase of firing distribution features displayed in Figure 2b,h correlated at the single cell level with the phase speed changes observed around interneuronal firing (Figure 2f,l), we first computed the spike-triggered phase speed change for each individual neuron. In doing so, we focused only on phase speed changes triggered by spikes in the up state, because only in this case enough spikes per neuron for a robust single-cell phase-speed-change analysis were observed. We then analyzed the correlation of the phase speed change of each cell with various different features of the phase of firing distribution in the up state. We found correlation between the single-cell phase speed change and the circular variance of the phase of firing distribution, which quantifies the width of the distribution, for both PV (correlation 0.67, p = 5E-3, N = 16) and SST interneurons (correlation 0.59, p = 3E-2, N = 14). Cells with particularly large distributions (circular variance ≥0.35) tended to show large speed change (Figure 2—figure supplement 4a). We also found correlation between single-cell phase speed changes and the preferred phase of firing for both PV (circular-linear correlation 0.62, p = 5E-2, N = 16) and SST cells (circular-linear correlation 0.74, p = 2E-2, N = 14). Cells with earlier preferred phase of firing tended to show larger speed change (Figure 2—figure supplement 4b). Preferred phase of firing and circular variance, however, tended to be correlated across cells (Figure 2—figure supplement 4c), making it difficult to dissociate the independent effect of each of these two variables on phase speed changes.

Altogether, these findings led us to hypothesize that the firing of both SST and PV interneurons during up states may causally contribute to their ending, whereas the firing of interneurons during down state, at least for PV cells, may delay the transition from the down state to the up state.

### Optogenetic activation of interneurons triggers up-to-down transitions

**Figure 3.**
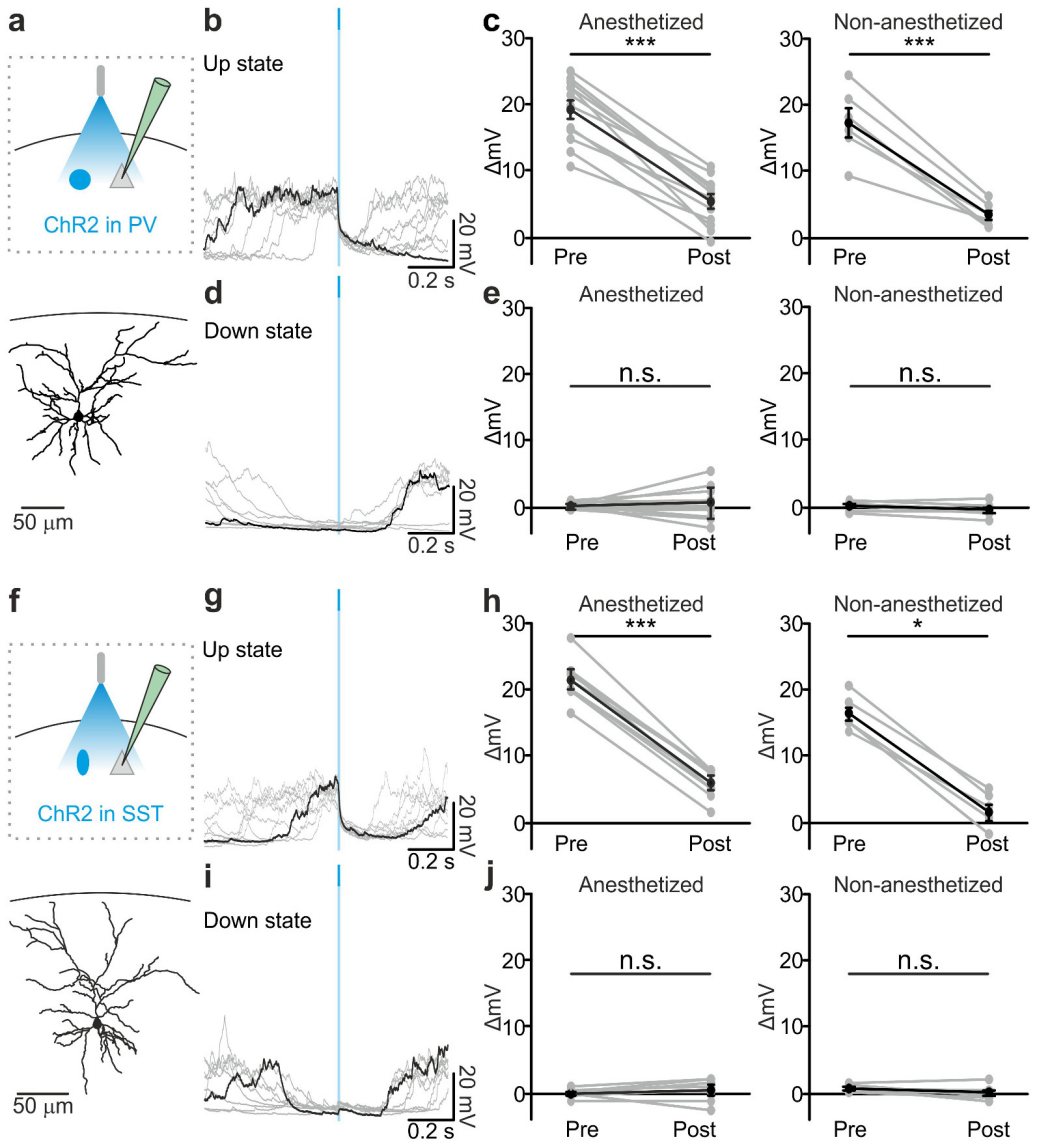
Optogenetic activation of PV and SST interneurons triggers up-to-down transitions. (**a**) Top: schematic of the experimental configuration. ChR2 is expressed in PV cells (blue round circle) and intracellular recordings are performed from pyramidal neurons (grey triangle). Bottom: morphological reconstruction of one recorded layer II/III pyramidal neuron. (**b**) Representative intracellular recordings showing the effect of PV interneuron activation when light (blue line) was delivered during an ongoing up state. Ten different trials are shown (one in black and the other in grey). (**c**) Change in the membrane potential of recorded cells (ΔmV) before (Pre) and after (Post) PV interneuron activation during an ongoing up state in anesthetized (left) and non-anesthetized (right) mice. Left, p = 7E-8, paired Student’s *t*-test, N = 12 cells from seven animals; right, p = 5E-4, paired Student’s *t*-test, N = 6 cells from three animals. (**d–e**) Same as in b-c but for PV activation during ongoing down states. In e: left, p = 5E-1, paired Student’s *t*-test, N = 12 cells seven animals; right, p = 7E-2, paired Student’s *t*-test, N = 6 cells from three animals. (**f–j**) Same as in a-e but for photoactivation of SST interneurons. In h: left, p = 1E-8, paired Student’s *t*-test, N = 9 cells from six animals; right, p = 3E-2, Wilcoxon signed rank test, N = 6 cells from three animals. In j: left, p = 2E-1, paired Student’s *t*-test, N = 9 cells from six animals; right, p = 2E-1, paired Student’s *t*-test, N = 6 cells from three animals. **DOI:** http://dx.doi.org/10.7554/eLife.26177.013 10.7554/eLife.26177.014Figure 3—source data 1.Source data for the analysis of membrane potential changes during photostimulation of PV or SST interneurons.**DOI:** http://dx.doi.org/10.7554/eLife.26177.014

To directly test whether interneurons causally contribute to up-to-down state transitions, we optogenetically activated PV and SST interneurons during spontaneous slow oscillations. Selective expression of ChR2 in PV and SST interneurons was achieved using AAV injections in PV-Cre and SST-Cre mice, respectively. Immunohistochemical (Figure 3—figure supplement 1) and electrophysiological (Figure 3—figure supplement 2) characterization confirmed the specificity of expression and functionality of the opsin. Using patch-clamp recording from layer II/III principal neurons in injected PV-Cre and SST-Cre mice under anaesthesia (Figure 3), we found that a brief (duration: 10 ms) light stimulus applied during an ongoing up state generated a pronounced and long-lasting hyperpolarization which outlasted the light stimulus and resembled a transition to a down state (Figure 3a–c, left panel, Figure 3f–h, left panel) but with larger slope compared to spontaneous events (Figure 3—figure supplement 3). All recordings were performed in the proximity of the illuminated area (see Materials and methods). When we observed the effect of the optogenetic manipulation at the network level using extracellular LFP and MUA recordings, we found similar results. Photoactivation of PV and SST cells during an ongoing up state resulted in a sudden decrease in the power of high-frequency oscillations in the LFP and in the reduction of the spike frequency in the MUA signal. Both effects outlasted the light stimulus and were compatible with a full transition to a down state (Figure 3—figure supplement 4a–e, Figure 3—figure supplement 5a–e). We then performed patch-clamp recordings in awake, head-restrained mice during quiet wakefulness. Under these experimental conditions cortical activities were characterized by frequent transitions between the up and the down states in accordance with previous reports (Petersen et al., 2003b). Importantly, we found (Figure 3c,h right panels and Figure 3—figure supplement 6) that the effect of optogenetic activation of PV and SST interneurons during an ongoing up state was similar to that observed in anesthetized mice, ruling out any possible side effect of anaesthesia.

**Figure 3—figure supplement 1.**
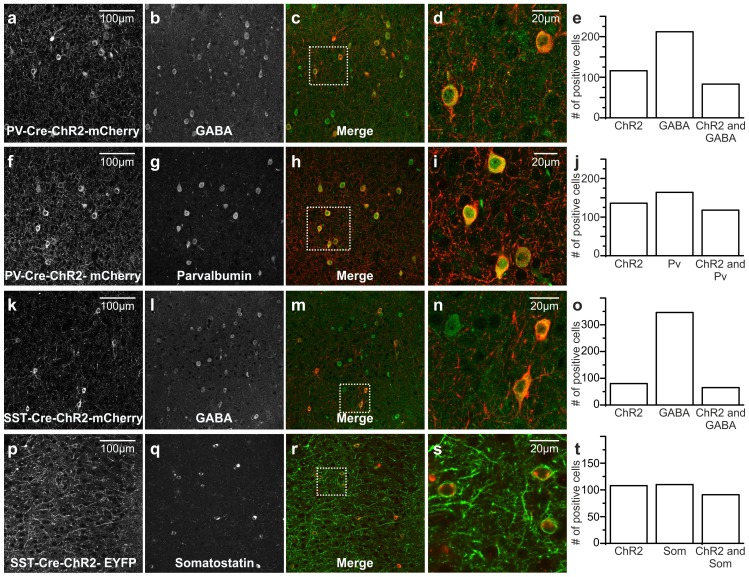
Immunohistochemical analysis of ChR2 positive cells in PV- and SST-Cre animals. (**a–d**) Confocal images of coronal cortical sections from a PV-Cre animal injected with AAV trasducing ChR2-mCherry. ChR2 positive cells (shown in a) largely stain for GABA (shown in b). ChR2-mCherry and GABA stainings are shown merged in c and the zoom of the region within the white square box is reported in d. (**e**) Total number of ChR2-positive, GABA-positive and ChR2 and GABA-positive neurons in PV-Cre injected mice (N = 4 animals, three sections per animal). (**f–i**) Same as in a-d but with a fluorescence immunostaining for parvalbumin. (**j**) Same as in e but for immunostaining for parvalbumin positive neurons (N = 4 animals, three sections per animal). (**k–t**) Same as in a-j but for SST-Cre animal injected with AAV trasducing ChR2-mCherry. In o, N = 4 animals, three sections per animal. In t, N = 4 animals, three sections per animal. **DOI:** http://dx.doi.org/10.7554/eLife.26177.015 10.7554/eLife.26177.016Figure 3—figure supplement 1—source data 1.Source data for the immunohistochemical analysis of ChR2 positive cells in PV-Cre and SST-Cre injected mice.**DOI:** http://dx.doi.org/10.7554/eLife.26177.016

**Figure 3—figure supplement 2.**
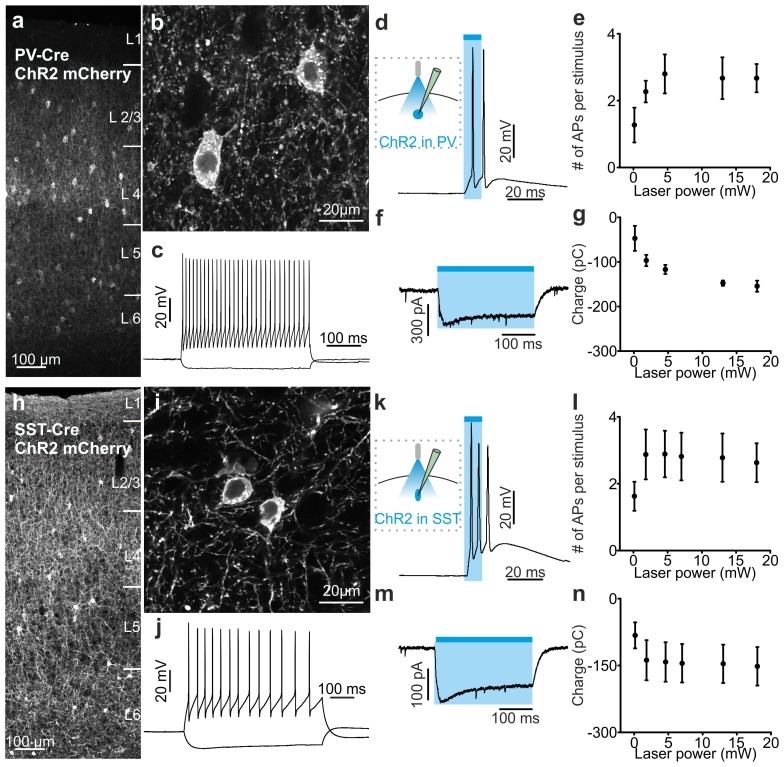
Functional analysis of PV and SST positive cells expressing ChR2. (**a–b**) Confocal images of one coronal cortical section showing the expression of ChR2-mCherry in PV-Cre mice. Cells are shown at an expanded time scale in b. (**c**) Representative current-clamp patch-clamp recordings in slices, showing the typical firing pattern of a PV cell positive for ChR2. (**d**) Left: schematic configuration for intracellular recordings from PV interneurons expressing ChR2 in coronal slices. Right: membrane depolarization and action potential (AP) firing in response to a 10 ms pulse of light. (**e**) Number of APs fired as a function of the light intensity (N = 5 cells from two animals). (**f**) Voltage-clamp patch-clamp recording showing the photocurrent evoked in response to 300 ms of light stimulus. (**g**) Quantification of the total amount of net charge passing through the cell membrane as a function of the light intensity (N = 3 cells from two animals). (**h–n**) Same as in a-g but for SST cells positive for ChR2. In l, N = 9 cells from three animals. In n, N = 5 cells from two animals. **DOI:** http://dx.doi.org/10.7554/eLife.26177.017 10.7554/eLife.26177.018Figure 3—figure supplement 2—source data 1.Source data for the functional characterization of PV and SST interneurons expressing ChR2.**DOI:** http://dx.doi.org/10.7554/eLife.26177.018

**Figure 3—figure supplement 3.**
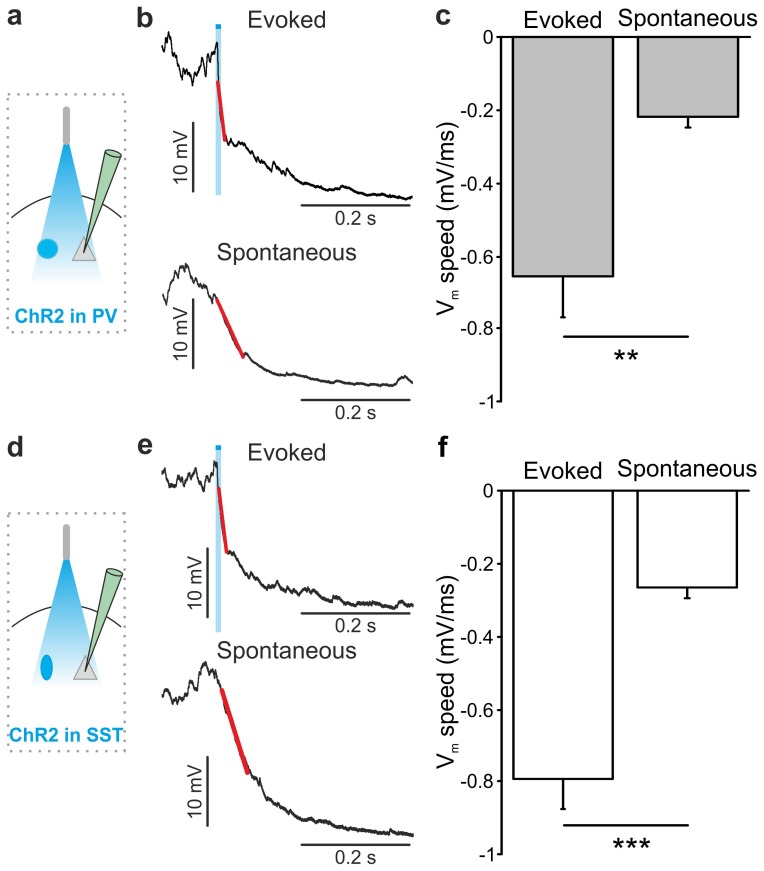
Membrane potential speed in pyramidal neurons during optogenetically-evoked and spontaneous up-to-down state transitions. (**a**) Schematic of the experimental configuration. (**b**) Representative intracellular recording showing an up-to-down state transition evoked by optogenetic activation (blue bar) of PV interneurons (top) or spontaneously occurring (bottom). A linear fit (red line) was used to evaluate membrane potential speed during the transition. (**c**) Average membrane potential speed in pyramidal neurons during optogenetically-triggered and spontaneous up-to-down state transitions (paired Student’s *t*-test, N = 12 cells from seven animals, p = 2E-3). (**d–f**) Same as in a-c but for optogenetic activation of SST interneurons (evoked *vs* spontaneous, paired Student’s *t*-test, N = 9 cells from six animals, p = 5E-4). The slope of optogenetically-evoked transitions was not significantly different when SST and PV cells were stimulated (SST *vs* PV, Student’s *t*-test, N = 9 cells and N = 12 respectively, p = 4E-1). **DOI:** http://dx.doi.org/10.7554/eLife.26177.019 10.7554/eLife.26177.020Figure 3—figure supplement 3—source data 1.Source data for the analysis of membrane potential speed during optogenetically-evoked and spontaneous up-to-down state transitions.**DOI:** http://dx.doi.org/10.7554/eLife.26177.020

**Figure 3—figure supplement 4.**
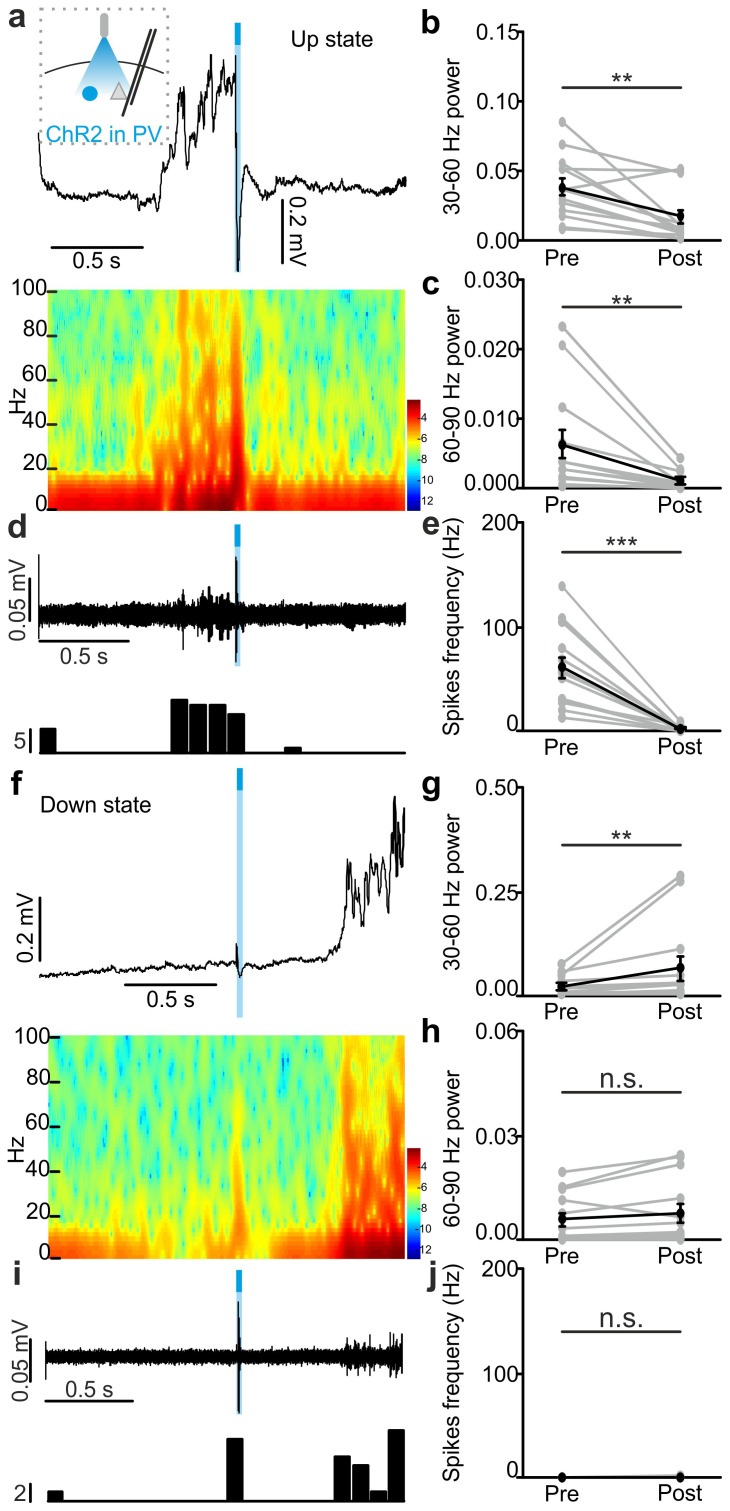
In vivo extracellular recordings of spontaneous cortical dynamics and photostimulation of PV positive interneurons expressing ChR2. (**a**) Representative trace of an in vivo LFP recording (top) and corresponding spectrogram (bottom) showing the effect of optogenetic activation of PV interneurons during an ongoing up state. The schematic of the experimental configuration is shown in the inset (top panel). (**b–c**) Average power in the 30–60 (**b**) and 60–90 (**c**) Hz frequency band before (Pre), and after (Post) light stimulation. Power values are normalized to the total power in the ‘Pre’ time window. In b, p = 2E-3, Wilcoxon signed-rank test, N = 13 animals. In c, p = 4E-3, Wilcoxon signed-rank test, N = 13 animals. (**d**) Top: multi-unit signal corresponding to the trace showed in a. Bottom: peri-stimulus time histogram (PSTH) of the trace showed in the top panel. (**e**) Average firing frequency of spikes (Hz) recorded in the multi-unit signal before (Pre) and after (Post) light stimulus (p = 2E-4, Wilcoxon signed-rank test, N = 13 animals). (**f–j**) Same as in a-e but for optogenetic activation of PV interneurons during an ongoing down state. In g, p = 5E-3, Wilcoxon signed-rank test, N = 13 animals. In h, p = 5E-2, Wilcoxon signed-rank test, N = 13 animals. In j, p = 3E-1, Wilcoxon signed-rank test, N = 13 animals. **DOI:** http://dx.doi.org/10.7554/eLife.26177.021 10.7554/eLife.26177.022Figure 3—figure supplement 4—source data 1.Source data for the effect of PV photoactivation during up states on network activity.**DOI:** http://dx.doi.org/10.7554/eLife.26177.022 10.7554/eLife.26177.023Figure 3—figure supplement 4—source data 2.Source data for the effect of PV photoactivation during down states on network activity.**DOI:** http://dx.doi.org/10.7554/eLife.26177.023

**Figure 3—figure supplement 5.**
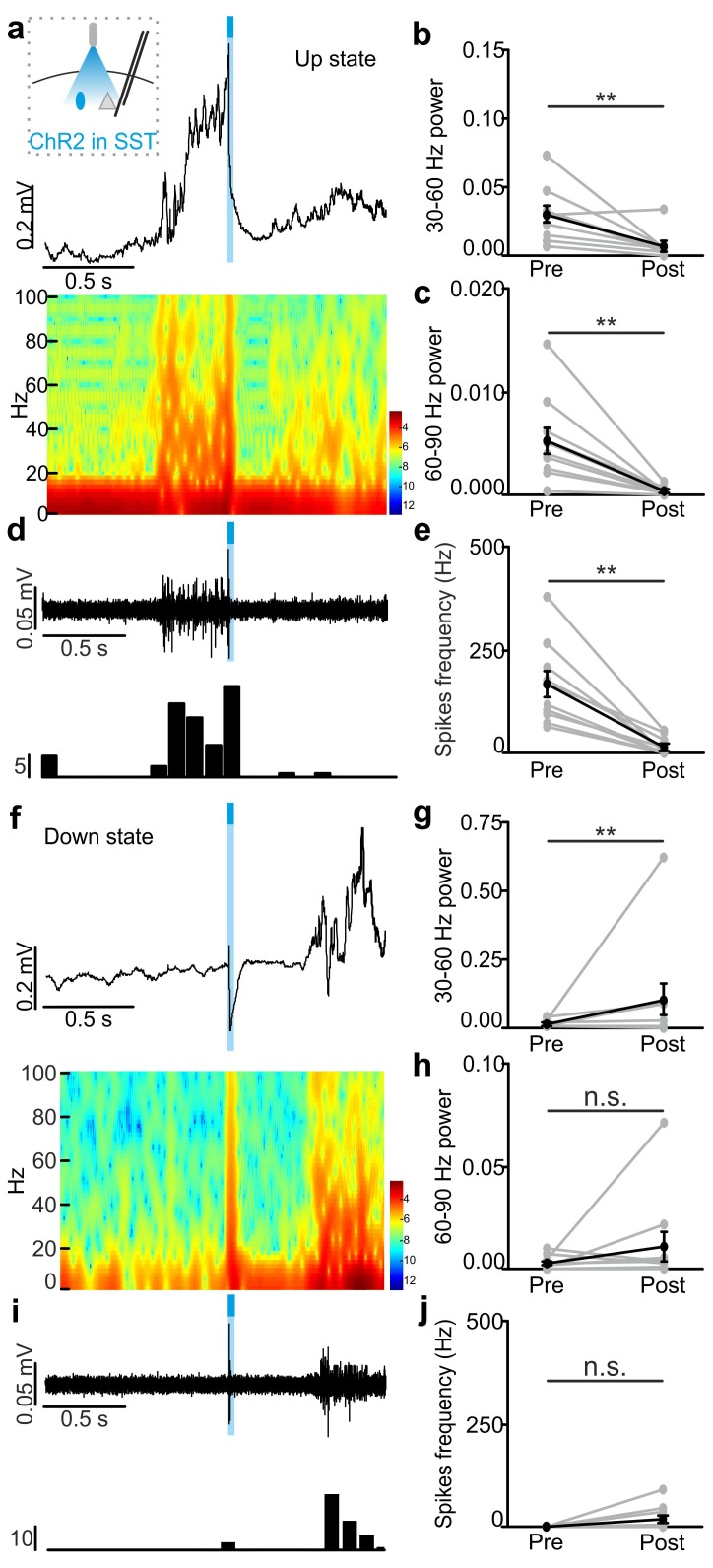
In vivo extracellular recordings of spontaneous cortical dynamics and photostimulation of SST positive interneurons expressing ChR2. (**a**) Representative trace of an in vivo LFP recording (top) and corresponding spectrogram (bottom) showing the effect of optogenetic activation of SST interneurons expressing ChR2 during an ongoing up state. The schematic of the experimental configuration is shown in the inset (top panel). (**b–c**) Average power in the 30–60 (**b**) and 60–90 (**c**) Hz frequency band before (Pre), and after (Post) light stimulation. Power values are normalized to the total power in the ‘pre’ time window. In b, p = 4E-3, Wilcoxon signed-rank test, N = 10 animals. In c, p = 9E-3, Wilcoxon signed-rank test, N = 10 animals. (**d**) Multi-unit signal (top) and corresponding PSTH (bottom) related to the trace showed in a. (**e**) Average firing frequency of spikes (Hz) recorded in the multi-unit signal before (Pre) and after (Post) light stimulus (p = 2E-3, Wilcoxon signed-rank test, N = 10 animals). (**f–j**) Same as in a-e but for optogenetic activation of SST interneurons during an ongoing down state. In g, p = 8E-3, Wilcoxon signed-rank test, N = 10 animals. In h, p = 6E-1, Wilcoxon signed-rank test, N = 10 animals. In j, p = 1E-1, Wilcoxon signed-rank test, N = 10 animals. **DOI:** http://dx.doi.org/10.7554/eLife.26177.024 10.7554/eLife.26177.025Figure 3—figure supplement 5—source data 1.Source data for the effect of SST photoactivation during up states on network activity.**DOI:** http://dx.doi.org/10.7554/eLife.26177.025 10.7554/eLife.26177.026Figure 3—figure supplement 5—source data 2.Source data for the effect of SST photoactivation during down states on network activity.**DOI:** http://dx.doi.org/10.7554/eLife.26177.026

**Figure 3—figure supplement 6.**
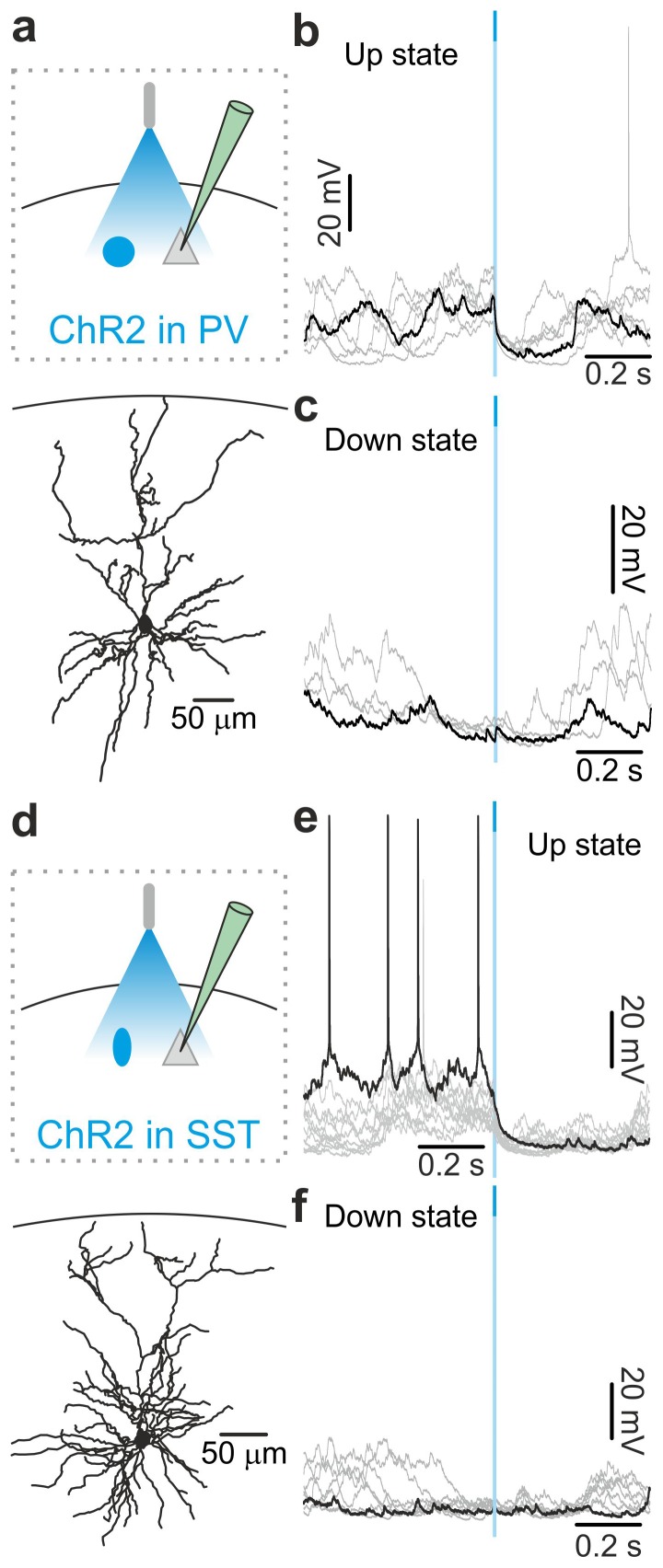
In vivo intracellular recordings of spontaneous cortical dynamics and photostimulation of PV or SST positive interneurons in non-anesthetized animals. (**a**) Top: schematic of the experimental configuration. Bottom: morphological reconstruction of a recorded layer II/III pyramidal neuron. (**b**) Representative intracellular recordings showing the effect of PV interneuron activation on the membrane potential of the recorded pyramidal neuron when light (blue line) was delivered during ongoing up states in a non-anesthetized mouse. (**c**) Same as in b but for photoactivation of PV interneurons during down states. (**d–f**) Same as in a-c but in mice expressing ChR2 in SST interneurons. **DOI:** http://dx.doi.org/10.7554/eLife.26177.027

Stimulation of PV and SST interneurons during a down state did not cause a significant change in the membrane potential of the recorded neuron in both anesthetized (Figure 3d,e left panel, Figure 3i,j left panel) and non-anesthetized mice (Figure 3e,j right panels and Figure 3—figure supplement 6). LFP and MUA experiments also confirmed no major effect of optogenetic activation of PV and SST interneuron during cortical downstate (Figure 3—figure supplement 4f–j; Figure 3—figure supplement 5f–j).

### Endogenous firing of interneurons controls up-to-down and down-to-up transitions

**Figure 4.**
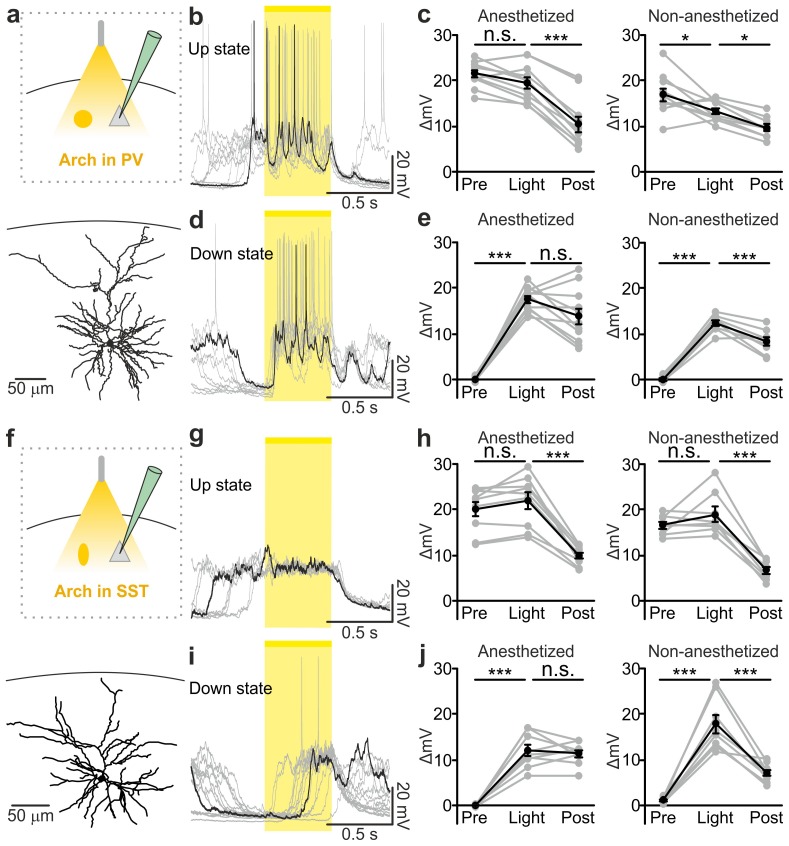
Optogenetic inhibition of PV and SST cells prolongs the up state and triggers down-to-up transitions. (**a**) Top: schematic of the experimental configuration. Bottom: morphological reconstruction of a representative recorded neuron. (**b**) Representative recordings from a pyramidal cell during optogenetic inhibition (yellow line) of PV interneurons during an up state. (**c**) Change in the membrane potential (ΔmV) of recorded neurons before (Pre), during (Light), and after (Post) optogenetic suppression of PV cells in anesthetized (left) and non-anesthetized (right) mice. Left: p = 3E-8, one-way ANOVA, N = 11 cells from four animals; right: p = 2E-8, one-way ANOVA, N = 10 cells from four animals. (**d–e**) Same as in b-c but for PV suppression during ongoing down states. In e: left, p = 7E-8, one-way ANOVA, N = 11 cells from four animals; right, p = 2E-6, one-way ANOVA, N = 8 cells from four animals. (**f–j**) Same as in a-e but for optogenetic inhibition of SST interneurons. In h: left, p = 1E-6, one-way ANOVA, N = 9 cells from five animals; right, p = 7E-7, one-way ANOVA, N = 8 cells from five animals. In j: left, p = 1E-6, one-way ANOVA, N = 9 cells from five animals; right, p = 2E-5, one-way ANOVA, N = 8 cells from five animals. **DOI:** http://dx.doi.org/10.7554/eLife.26177.028 10.7554/eLife.26177.029Figure 4—source data 1.Source data for the analysis of membrane potential changes during photoinhibition of PV or SST interneurons.**DOI:** http://dx.doi.org/10.7554/eLife.26177.029

Optogenetic stimulation elicits APs in ChR2-positive cells in a way that may not fully recapitulate physiological firing patterns of cortical interneurons. To demonstrate that *endogenous* spiking activity of interneurons controls up-to-down transitions, we expressed the inhibitory opsin Archaerhodopsin (Chow et al., 2010) (Arch) in either PV or SST cells. We first controlled that Arch efficiently suppressed APs in PV (Figure 4—figure supplement 1a–e) and SST (Figure 4—figure supplement 1f–j) interneurons. We then delivered yellow light stimuli (λ = 594 nm; duration, 500 ms) during up states in anesthetized and non-anesthetized (Figure 4 and Figure 4—figure supplement 2) mice. Optogenetic inhibition of PV and SST interneurons prolonged the up state for the whole duration of the light stimulation, resulting in membrane potential depolarization in single pyramidal neurons (Figure 4a–c, and Figure 4f–h and Figure 4—figure supplement 2b,e). Moreover, optogenetic inhibition of PV and SST cells caused a significant increase in the gamma frequency power and spike frequency in the LFP and MUA signal, respectively (for PV cells Figure 4—figure supplement 3a–e; for SST interneurons, Figure 4—figure supplement 4a–e), events that were compatible with a prolongation of the up state by the optical manipulation. After stimulus offset, we observed a significant hyperpolarization of the membrane potential (Figure 4c,h) that resembled a transition to the down state in patch-clamp recordings in both PV and SST mice. In extracellular recordings, the stimulus end was associated with a decrease in the power of high-frequency oscillations in the LFP (Figure 4—figure supplement 3b,c, Figure 4—figure supplement 4b,c) and of the MUA signal (Figure 4—figure supplement 3e; Figure 4—figure supplement 4e), results which were again compatible with an up-to-down state transition at the end of the light stimulus.

**Figure 4—figure supplement 1.**
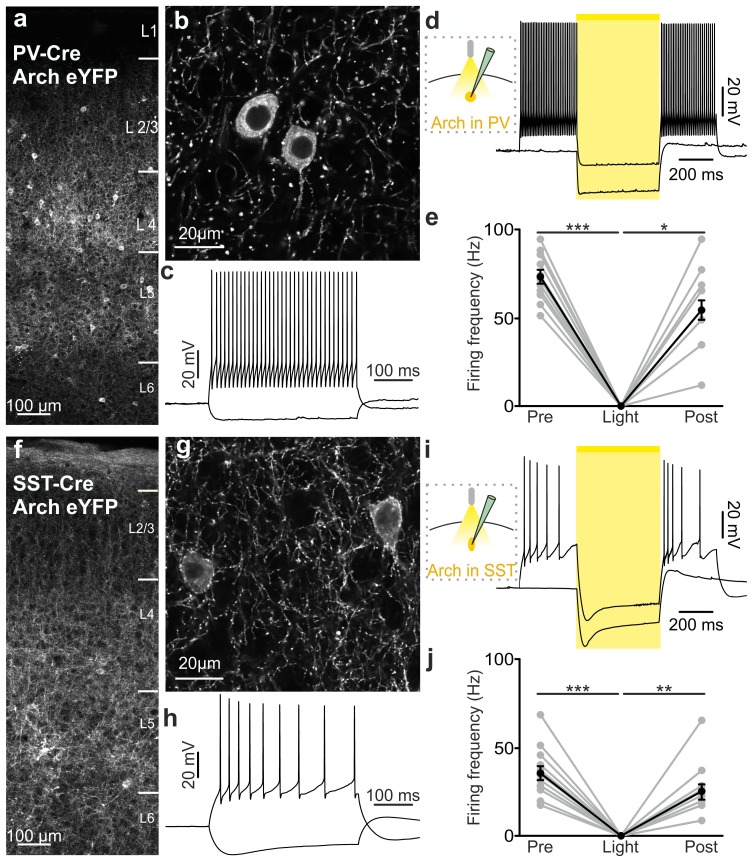
Functional analysis of PV and SST positive cells expressing Arch. (**a–b**) Confocal images of coronal cortical sections showing the expression of Arch-eYFP in PV-Cre mice. (**c**) Representative current-clamp patch-clamp recordings in slices, showing the typical firing pattern of a PV cell that was also positive for Arch. (**d**) Left: schematic of the experimental configuration. Right: membrane potential response to 500 ms of light stimulation. AP discharge was induced by current injection. (**e**) Quantification of the average firing frequency before (Pre), during (Light), and after (Post) light stimulation (p = 9E-6, Friedman test, N = 13 cells from four animals). (**f–j**) Same as in a-e but for SST positive interneurons that express Arch. In j, p = 2E-5, Friedman test, N = 13 cells from six animals. **DOI:** http://dx.doi.org/10.7554/eLife.26177.030 10.7554/eLife.26177.031Figure 4—figure supplement 1—source data 1.Source data for functional characterization of PV and SST interneurons expressing Arch.**DOI:** http://dx.doi.org/10.7554/eLife.26177.031

**Figure 4—figure supplement 2.**
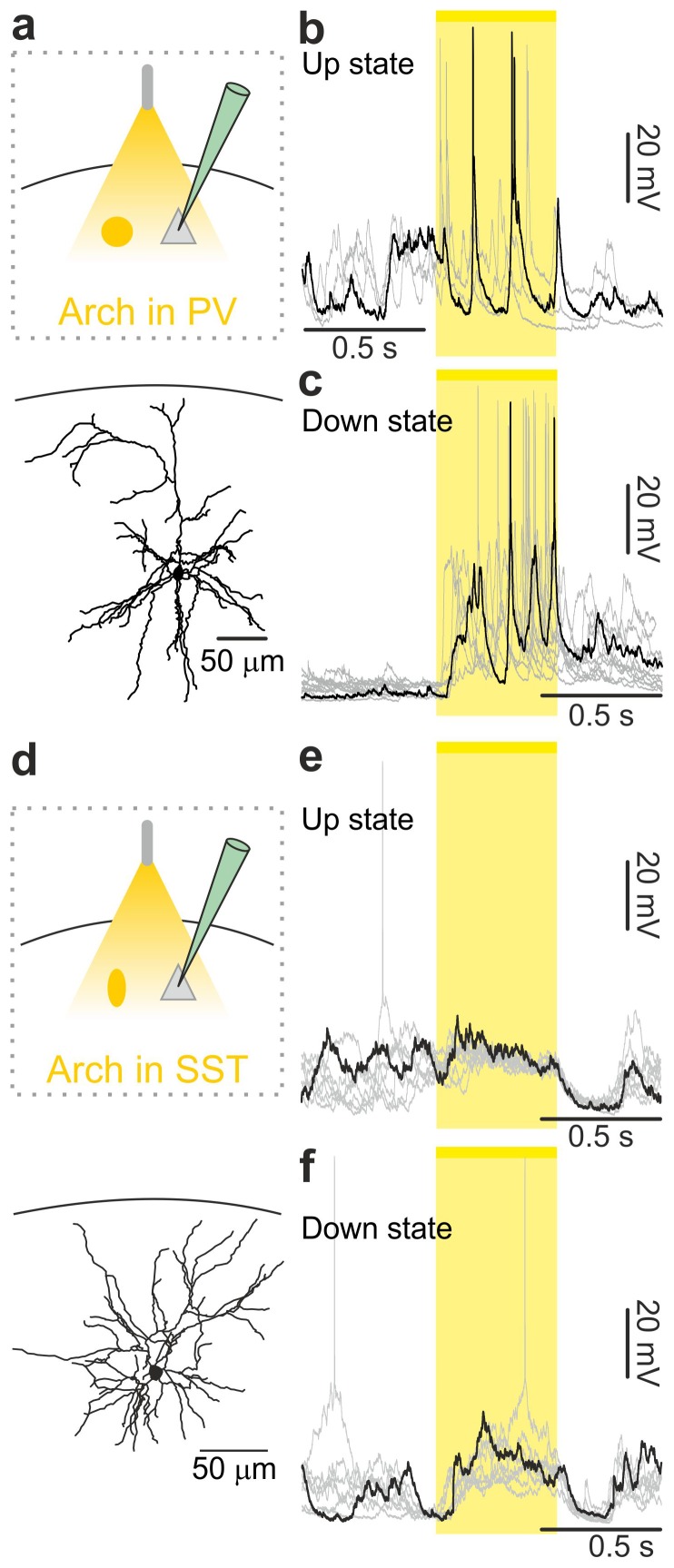
In vivo intracellular recordings of spontaneous cortical dynamics and photoinhibition of PV or SST positive interneurons in non-anesthetized animals. (**a**) Top: schematic representation of the experimental configuration. Bottom: morphological reconstruction of a recorded layer II/III pyramidal neuron. (**b**) Representative intracellular recordings showing the effect of the optogenetic inhibition of PV interneurons on the membrane potential of the recorded pyramidal neuron when light (yellow line and shadow) was delivered during up states in a non-anesthetized mouse. (**c**) Same as in b but for optogenetic inhibition of PV interneurons during down states. (**d–f**) Same as in a-c but in mice expressing Arch in SST interneurons. **DOI:** http://dx.doi.org/10.7554/eLife.26177.032

**Figure 4—figure supplement 3.**
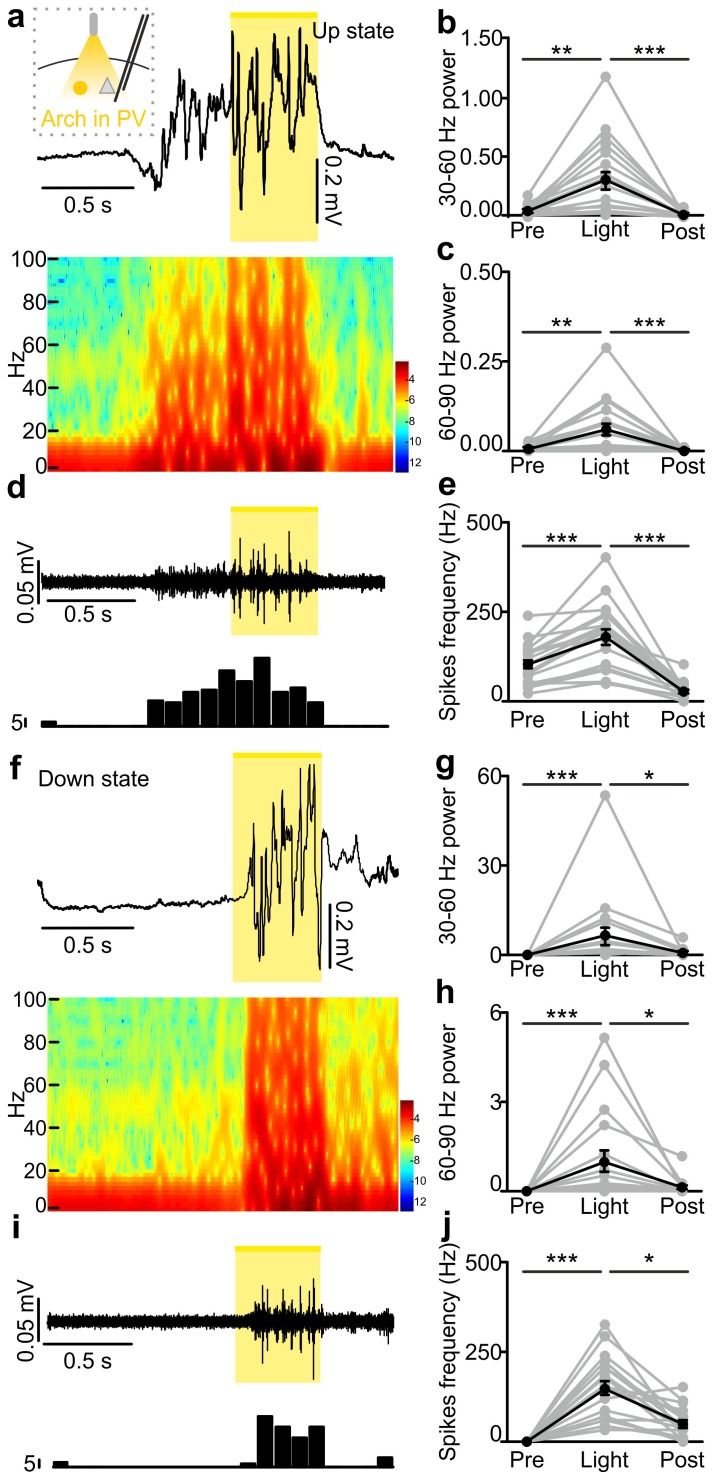
In vivo extracellular recordings of spontaneous cortical dynamics and photoinhibition of PV positive interneurons expressing Arch. (**a**) Representative trace of an in vivo LFP recording (top) and corresponding spectrogram (bottom) showing the effect of optogenetic stimulation (yellow line) of PV interneurons expressing Arch during an ongoing up state. The schematic of the experimental configuration is shown in the inset (top panel). (**b–c**) Average power in the 30–60 (**b**) and 60–90 (**c**) Hz frequency band before (Pre), during (Light) and after (Post) light stimulation. Power values are normalized to the total power in the ‘pre’ time window. In b, p = 1E-7, Friedman test, N = 19 animals. In c, p = 9E-9, Friedman test, N = 19 animals. (**d**) Multi-unit signal (top) and corresponding PSTH (bottom) related to the trace showed in a. (**e**) Average firing frequency of spikes (Hz) recorded in the multiunit signal before (Pre), during (Light), and after (Post) light stimulus (p = 1E-7, one-way ANOVA, N = 19 animals). (**f–j**) Same as in a-e but for photoinhibition of PV positive interneurons during an ongoing down state. In g, p = 1E-8, Friedman test, N = 19 animals. In h, p = 2E-8, Friedman test, N = 19 animals. In j, p = 3E-8, Friedman test, N = 19 animals. **DOI:** http://dx.doi.org/10.7554/eLife.26177.033 10.7554/eLife.26177.034Figure 4—figure supplement 3—source data 1.Source data for the effect of PV photoinhibition during up states on network activity.**DOI:** http://dx.doi.org/10.7554/eLife.26177.034 10.7554/eLife.26177.035Figure 4—figure supplement 3—source data 2.Source data for the effect of PV photoinhibition during down states on network activity.**DOI:** http://dx.doi.org/10.7554/eLife.26177.035

**Figure 4—figure supplement 4.**
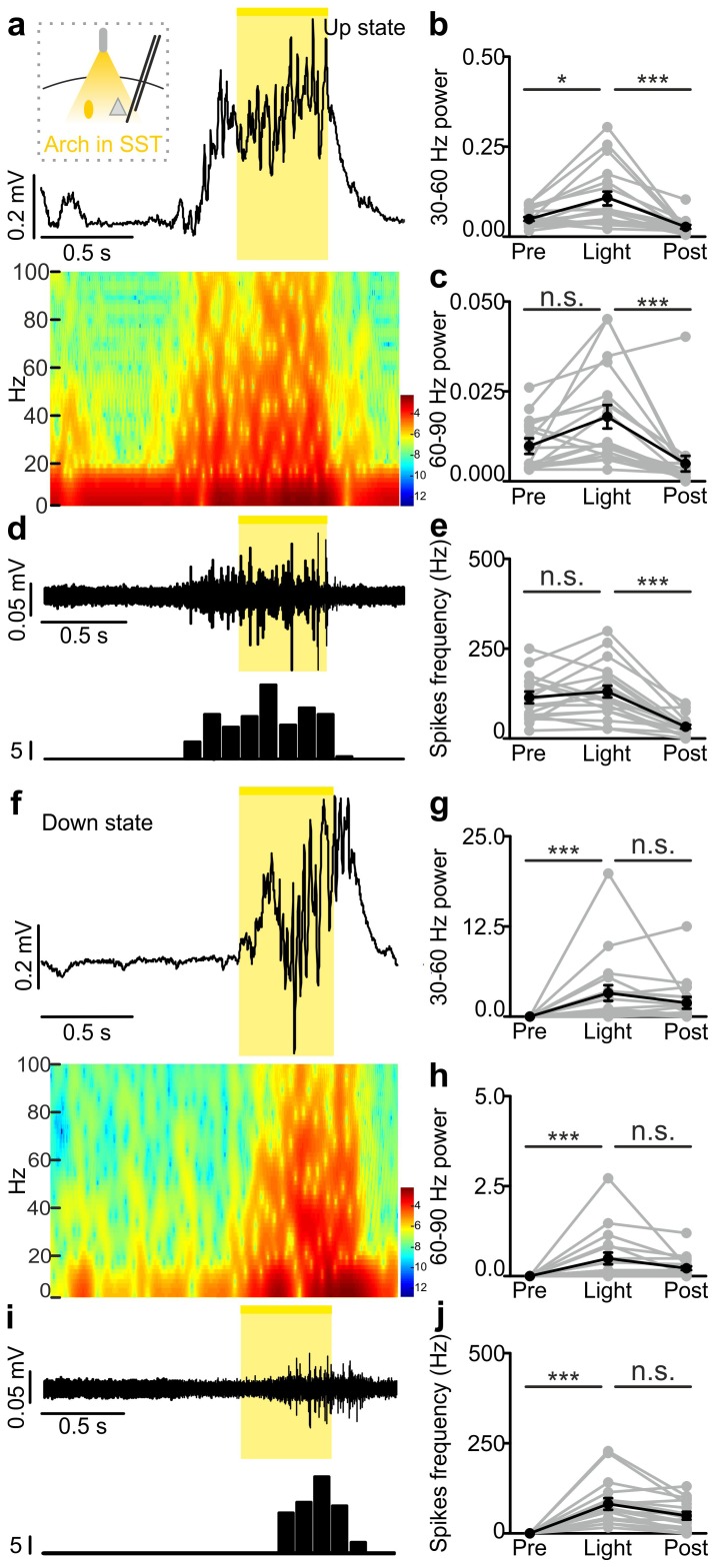
In vivo extracellular recordings of spontaneous cortical dynamics and photoinhibition of SST positive interneurons expressing Arch. (**a**) Representative trace of an in vivo LFP recording (top) and corresponding spectrogram (bottom) showing the effect of optogenetic stimulation (yellow line) of SST interneurons expressing Arch during an ongoing up state. The schematic of the experimental configuration is shown in the inset (top panel). (**b–c**) Average power in the 30–60 (**b**) and 60–90 (**c**) Hz frequency band before (Pre), during (Light), and after (Post) light stimulation. In b, p = 3E-7, Friedman test, N = 18 animals. In c, p = 3E-6, Friedman test, N = 18 animals. (**d**) Multi-unit signal (top) and corresponding PSTH (bottom) related to the trace showed in a. (**e**) Average firing frequency of spikes (Hz) recorded in the multi-unit signal before (Pre), during (Light), and after (Post) light stimulus (p = 4E-6, Friedman test, N = 18 animals). (**f–j**) Same as in a-e but for optogenetic inhibition of SST interneurons during an ongoing down state. In g, p = 1E-6, Friedman test, N = 18 animals. In h, p = 3E-7, Friedman test, N = 18 animals. In j, p = 3E-7, Friedman test, N = 18 animals. **DOI:** http://dx.doi.org/10.7554/eLife.26177.036 10.7554/eLife.26177.037Figure 4—figure supplement 4—source data 1.Source data for the effect of SST photoinhibition during up states on network activity.**DOI:** http://dx.doi.org/10.7554/eLife.26177.037 10.7554/eLife.26177.038Figure 4—figure supplement 4—source data 2.Source data for the effect of SST photoinhibition during down states on network activity.**DOI:** http://dx.doi.org/10.7554/eLife.26177.038

PV and SST interneurons fire preferentially during cortical up states (Puig et al., 2008; Tahvildari et al., 2012; Neske et al., 2015). However, our data (Figure 1c,h) showed that PV and SST interneurons fire also during down states and the bottom panel of Figure 2f suggests that the low firing activity of PV cells during the down state may influence down-to-up transition probability. To test this hypothesis we inhibited PV and SST cells during ongoing down states. We found that individual principal cells and the cortical network reliably transitioned to an active state, which resembled an up state, upon photoinhibition of interneurons. Optogenetic inhibition of PV and SST cells during an ongoing down state triggered a membrane depolarization in pyramidal cells in anesthetized and non-anesthetized mice (Figure 4d,e,i,j and Figure 4—figure supplement 2c,f) which could overlast the light stimulus. When looking at the cortical network activity with extracellular recordings, optogenetic inhibition of interneurons during an ongoing down state increased the power of high frequency oscillations in the LFP (PV inhibition, Figure 4—figure supplement 3f–h; SST inhibition, Figure 4—figure supplement 4f–h) and the MUA signal (PV inhibition, Figure 4—figure supplement 3i,j; SST inhibition, Figure 4—figure supplement 4i,j), effects which were compatible with a full network transition to an up state.

### Inhibitory control of state transition is interneuron subtype-specific

**Figure 5.**
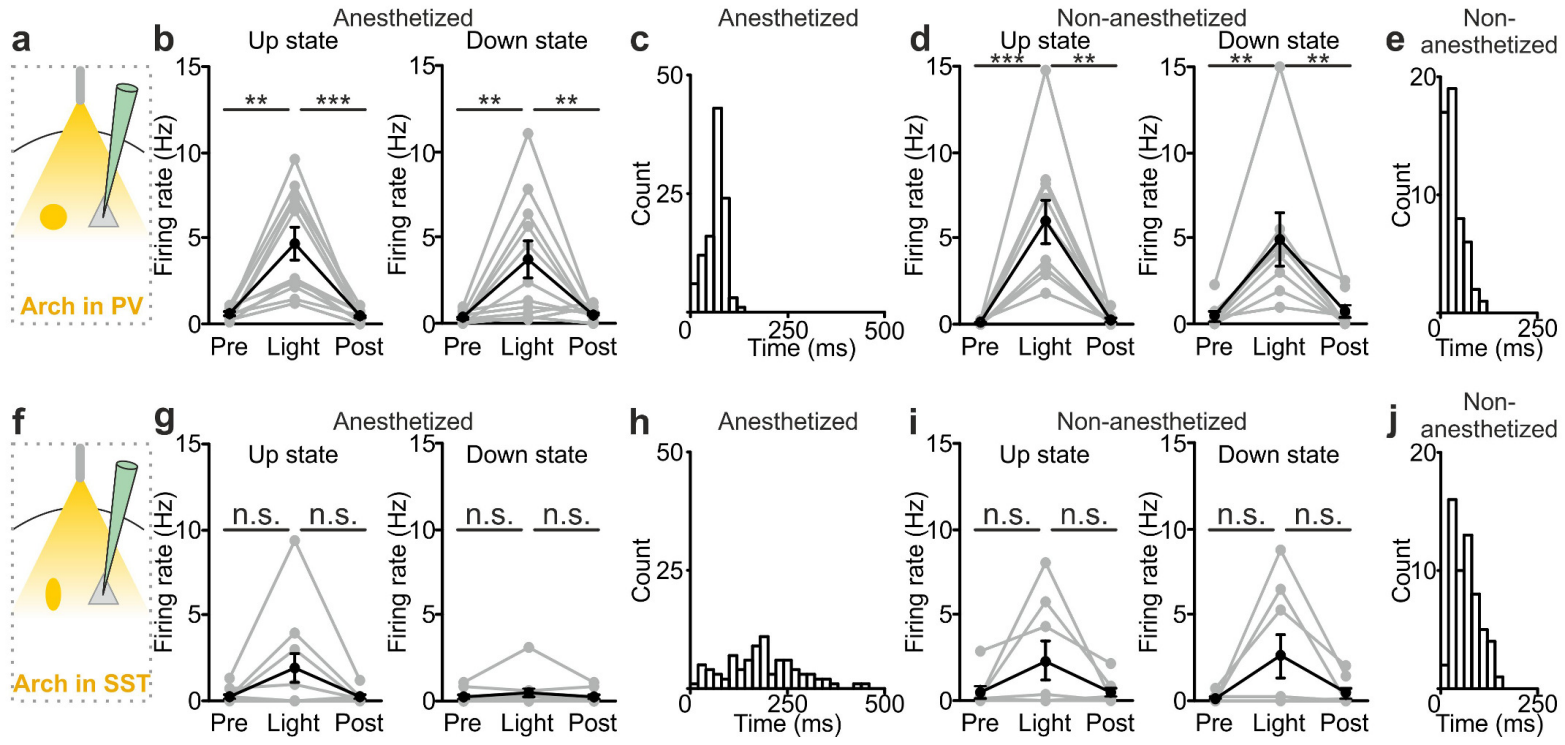
Interneuron type-specific effect of optogenetic inhibitory manipulations. (**a**) Schematic of the experimental configuration where PV cells were photoinhibited. (**b**) Firing rate of pyramidal neurons before (Pre), during (Light), and after (Post) PV photoinhibition during up (left) or down (right) state in anesthetized mice. Left: p = 2E-4, Friedman test, N = 11 cells from four animals; right: p = 1E-3, one-way ANOVA, N = 11 cells from four animals. (**c**) Distribution of the latency of the down-to-up state transition triggered by photoinhibition of PV cells during an ongoing down state in anesthetized mice. (**d–e**) Same as in b-c but in non-anesthetized mice. In d: left, p = 2E-4, Friedman test, N = 10 cells from four animals; right, p = 3E-4, Friedman test, N = 8 cells from four animals. (**f**) Schematic of the experimental configuration where SST interneurons were photoinhibited. (**g**) Same as in b but during photoinhibition of SST interneurons. Left: p = 6E-1, Friedman test, N = 9 cells from five animals; right: p = 2E-1, Friedman test, N = 9 cells from five animals. (**h**) Distribution of the latency of the down-to-up state transition triggered by photoinhibition of SST during an ongoing down state in anesthetized mice. (**i–j**) Same as in g-h but in non-anesthetized mice. In i: left, p = 7E-1, Friedman test, N = 8 cells from five animals; right, p = 5E-1, Friedman test, N = 8 cells from five animals. **DOI:** http://dx.doi.org/10.7554/eLife.26177.039 10.7554/eLife.26177.040Figure 5—source data 1.Source data for the analysis of the interneuron type-specific effect of optogenetic inhibitory manipulations.**DOI:** http://dx.doi.org/10.7554/eLife.26177.040

The previous experiments show that optogenetic inhibition of both PV and SST interneurons prolonged the up state and favoured down-to-up transitions. However, important differences between the effects of the manipulations of the two interneuron types were observed (Figure 5). First, suppression of PV, but not SST cells, during both up and down states significantly enhanced spiking activity of principal cells (Figure 5a,b,d). In both anesthetized and non-anesthetized animals, photoinhibition of SST interneurons during up states tended to increase the firing frequency of principal neurons, but the effect did not reach statistical significance (Figure 5f,g,i). Second, photoinhibition of PV cells during spontaneous down states generated up state transitions with shorter latency from the onset of the light stimulus compared to the photoinhibition of SST cells in both anesthetized (Figure 5c,h: average latency values: 69 ± 4 ms *vs* 181 ± 15 ms for PV and SST, respectively; p = 6E-5, unpaired Student’s *t*-test, PV N = 11 cells from four animals and SST N = 9 cells from five animals) and non-anesthetized mice (Figure 5e,j: average latency values: 30 ± 5 ms *vs* 67 ± 11 ms for PV and SST, respectively p = 9E-3 unpaired Student’s *t* -test, N = 8 cells from five animals for PV and 8 cells from five animals for SST). Third, inhibiting PV interneurons facilitated down-to-up transitions with less jitter compared to the inhibition of SST cells (in anesthetized mice, 20 ± 3 ms for PV inhibition *vs* 109 ± 17 ms for SST inhibition, p = 6E-4, unpaired Student’s *t*-test, N = 11 PV cells from four animals and N = 9 SST cells from five animals; in non-anesthetized mice, 16.8 ± 2.9 ms for PV inhibition *vs* 32.7 ± 6.1 ms for SST inhibition, p = 3E-2, unpaired Student’s *t*-test, N = 8 cells from five animals for PV and SST interneurons). Fourth, photoinhibition of PV cells triggered down-to-up transitions with larger slope compared to spontaneous transitions observed in the same recorded cell. In contrast, photosuppression of SST cells triggered down-to-up transitions with slopes that were indistinguishable from those of spontaneous down-to-up transitions (Figure 5—figure supplement 1).

**Figure 5—figure supplement 1.**
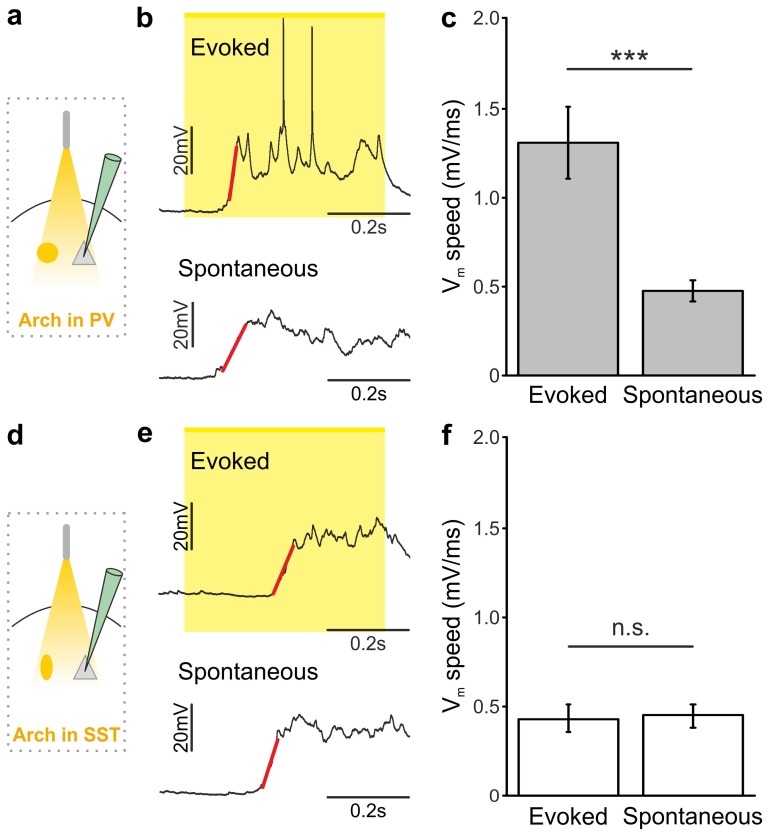
Membrane potential speed in pyramidal neurons during optogenetically-evoked and spontaneous down-to-up state transitions. (**a**) Schematic of the experimental configuration. (**b**) Representative intracellular recording showing a down-to-up state transition evoked by optogenetic inhibition (yellow bar) of PV interneurons (top) or spontaneously occurring (bottom). A linear fit (red line) was used to evaluate membrane potential speed during the transition. (**c**) Average membrane potential speed in pyramidal neurons during optogenetically-triggered and spontaneous down-to-up state transitions (paired Student’s *t*-test, N = 11 cells from four animals, p = 3E-4). (**d–f**) Same as in a-c but for optogenetic inhibition of SST interneurons (paired Student’s *t*-test, N = 9 cells from five animals, p = 7E-1). **DOI:** http://dx.doi.org/10.7554/eLife.26177.041 10.7554/eLife.26177.042Figure 5—figure supplement 1—source data 1.Source data for the analysis of the membrane potential speed during optogenetically-evoked and spontaneous down-to-up state transitions.**DOI:** http://dx.doi.org/10.7554/eLife.26177.042

### Local optogenetic manipulation of interneurons induces mesoscale state transitions

Our results showed that optogenetic perturbation of cortical interneurons control state transitions in cortical cells and networks located in proximity to the illuminated area. Given that up and down state transitions are known to occur over large cortical areas, we asked whether local optogenetic perturbation of interneurons could also affect cortical regions far from the illuminated site. To address this question, we first simultaneously recorded the MUA in two cortical regions that were approximately 2 mm apart in anesthetized mice. The two electrodes (Ch1 and Ch2, Figure 6—figure supplement 1) were lowered to the same cortical depth (~350 µm from the pial surface), and light was delivered to the more rostral recording site (Ch1) using a fiber optic positioned normal to the brain surface. In mice expressing ChR2 in PV cells, a short (duration: 10 ms) light stimulus significantly reduced the MUA signal at both channels (Ch1 and Ch2, Figure 6—figure supplement 1a–e). Similar results were observed in mice expressing ChR2 in SST interneurons (Figure 6—figure supplement 1f–j). To exclude the possibility that the effect on the MUA signal recorded in Ch2 (i.e., the electrode placed in the most caudal area) was due to direct illumination from the optical fiber placed near the rostral electrode (Ch1), we repeated these experiments using a patterned illumination system using a digital micromirror device (DMD) to precisely control the size of the illumination area. Consistent to what was previously observed, we found that the activation of PV or SST in a confined area (area diameter, 200 µm) projected close to Ch1 similarly affected network activities in both recording sites, resulting in a significant reduction of spike frequency in the MUA signal recorded at Ch1 and Ch2 (Figure 6—figure supplement 2).

**Figure 6.**
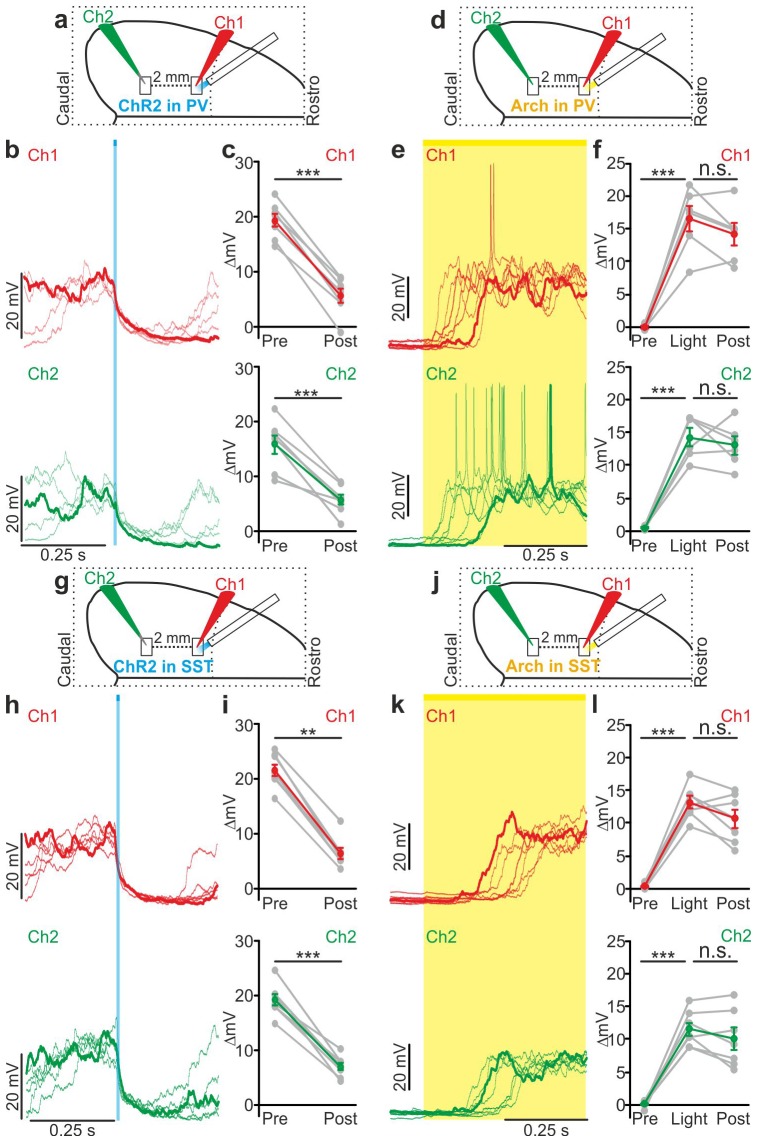
Local modulation of cortical interneurons causes large-scale state transitions. (**a**) Schematic representation of the experimental configuration. Simultaneous dual patch-clamp recordings were performed in anesthetized mice during photoactivation of PV interneurons expressing ChR2: Ch1 (red) indicates the recording site located close to the illuminated area, whereas Ch2 (green) represents the recording site placed 2 mm away from Ch1 in the caudal direction. (**b**) Representative traces of two simultaneously recorded neurons (top, Ch1, red trace; bottom, Ch2 green trace) during photoactivation of PV interneurons. Bold red (Ch1) and green (Ch2) lines show a single representative trial. (**c**) Change in the membrane potential (ΔmV) of recorded neurons before (Pre) and after (Post) PV activation during an ongoing up state. Grey dots and lines indicate single experiments, red (Ch1) or green (Ch2) dots and lines indicate the average value represented as mean ± s.e.m. Top: p = 7E-6, paired Student’s *t*-test, N = 7 cells from three animals; bottom: p = 6E-4, paired Student’s *t*-test, N = 7 cells from three animals. (**d**) Schematic of the experimental configuration for dual patch-clamp recordings during photoinhibition of PV interneurons. (**e**) Representative traces from two simultaneously recorded neurons (Ch1, red; Ch2, green) when photoinhibition of PV interneurons occurred during cortical down states. (**f**) Change in the membrane potential (ΔmV) of recorded neurons before (Pre), during (Light), and after (Post) photoinhibition of PV cells. Top: p = 4E-4, one-way ANOVA, N = 6 cells from four animals; bottom: p = 4E-5, one-way ANOVA, N = 6 cells from four animals. (**g**) Schematic representation of the experimental configuration for dual-patch clamp recording in mice expressing ChR2 in SST interneurons. (**h–i**) Same as in b-c but during photoactivation of SST interneurons. In i: top, p = 8E-3, Wilcoxon signed-rank test, N = 8 cells from six animals; bottom, p = 7E-6, paired Student’s *t*-test, N = 8 cells from six animals. (**j**) Schematic representation of the experimental configuration for paired patch-clamp recording in mice expressing Arch in SST interneurons. (**k–l**) Same as in h-i but inhibiting SST interneurons. In l: top, p = 3E-5, one-way ANOVA, N = 7 cells from four animals; bottom, p = 1E-4, one-way ANOVA, N = 7 cells from four animals. **DOI:** http://dx.doi.org/10.7554/eLife.26177.043 10.7554/eLife.26177.044Figure 6—source data 1.Source data for the analysis of membrane potential changes in simultaneously recorded neurons during local optogenetic perturbation of PV and SST interneurons.**DOI:** http://dx.doi.org/10.7554/eLife.26177.044

We then tested the effect of local interneurons modulation on the membrane potential of cortical neurons located far from the modulated region. To this aim, we performed dual patch-clamp recordings from superficial pyramidal neurons in anesthetized (Figure 6) mice. We found that light stimulation induced significant membrane potential hyperpolarization in both recorded neurons (Figure 6a–c,g–i), efficiently driving cells from the up to the down state in both PV and SST mice expressing ChR2 (PV: response delay between Ch2 and Ch1, 3.2 ± 0.6 ms; SST: response delay between Ch2 and Ch1, 3.5 ± 1.1 ms, Figure 6—figure supplement 3a–b). Similar results were obtained in awake animals when recording from single pyramidal neurons located ~2 mm apart from the stimulated area (Figure 6—figure supplement 4a–c,g–i). Finally, we extended this experimental design to mice expressing Arch in PV or SST interneurons. Photoinhibition of PV or SST interneurons in anesthetized animals enhanced network activity and significantly increased the MUA signal during light stimulation at both recording sites (Figure 6—figure supplement 5). Moreover, in paired recording experiments in anesthetized mice we found that photoinhibition of PV or SST significantly facilitated down-to-up transitions in both recorded neurons (Figure 6d–f,j–l) with variable response delays (Figure 6—figure supplement 3c–d). This was confirmed in awake non-anesthetized animals, where we observed a significant membrane potential depolarization during light stimulation in single recorded neurons located far (~2 mm) from the illuminated area (Figure 6—figure supplement 4d–f,j–l).

**Figure 6—figure supplement 1.**
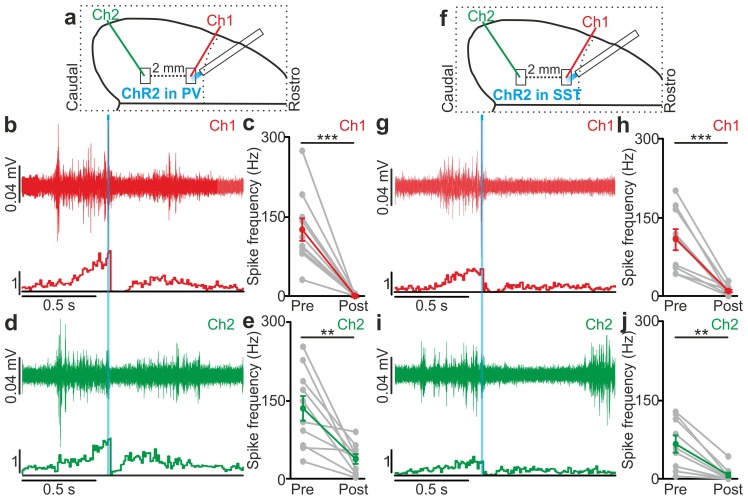
Optogenetic activation of interneurons modulates network multi-unit activity over large cortical territories in anesthetized animals. (**a**) Schematic representation of the experimental setup. Simultaneous extracellular recordings are performed in anesthetized mice during photoactivation of PV interneurons expressing ChR2. In this as well in other figures, Ch1 (red) indicates the recording site located close to the illuminated area, whereas Ch2 (green) represents the recording site placed 2 mm away from Ch1 in the caudal direction. (**b**) Top: example of a multi-unit signal recorded in Ch1 during optogenetic activation of PV interneurons (blue line). Bottom: average PSTH of Ch1 for all recorded animals (N = 10). (**c**) Average frequency of spikes recorded in Ch1 before (Pre) and after (Post) light stimulation (p = 3E-4, paired Student’s *t*-test, N = 10 animals). In this as well in other figures: grey dots and lines in this type of graph indicate single experiments, red dots and lines indicate the average value represented as mean ± s.e.m. (**d**) Top: multi-unit signal recorded in Ch2 simultaneously with the signal in Ch1 show in b (top panel). Bottom: average PSTH of Ch2 for all recorded animals (N = 10). (**e**) Same as in c but for recordings in Ch2 (p = 3E-3, paired Student’s *t*-test, N = 10 animals). Green dots and lines indicate the average value represented as mean ± s.e.m. (**f–j**) Same as in a–e but during photostimulation of SST interneurons expressing ChR2. In h, p = 9E-4, paired Student’s *t*-test, N = 9 animals. In j, p = 4E-3, Wilcoxon signed-rank test, N = 9 animals. **DOI:** http://dx.doi.org/10.7554/eLife.26177.045 10.7554/eLife.26177.046Figure 6—figure supplement 1—source data 1.Source data for the effect of local PV activation on network activity over large cortical territories.**DOI:** http://dx.doi.org/10.7554/eLife.26177.046 10.7554/eLife.26177.047Figure 6—figure supplement 1—source data 2.Source data for the effect of local SST activation on network activity over large cortical territories.**DOI:** http://dx.doi.org/10.7554/eLife.26177.047

**Figure 6—figure supplement 2.**
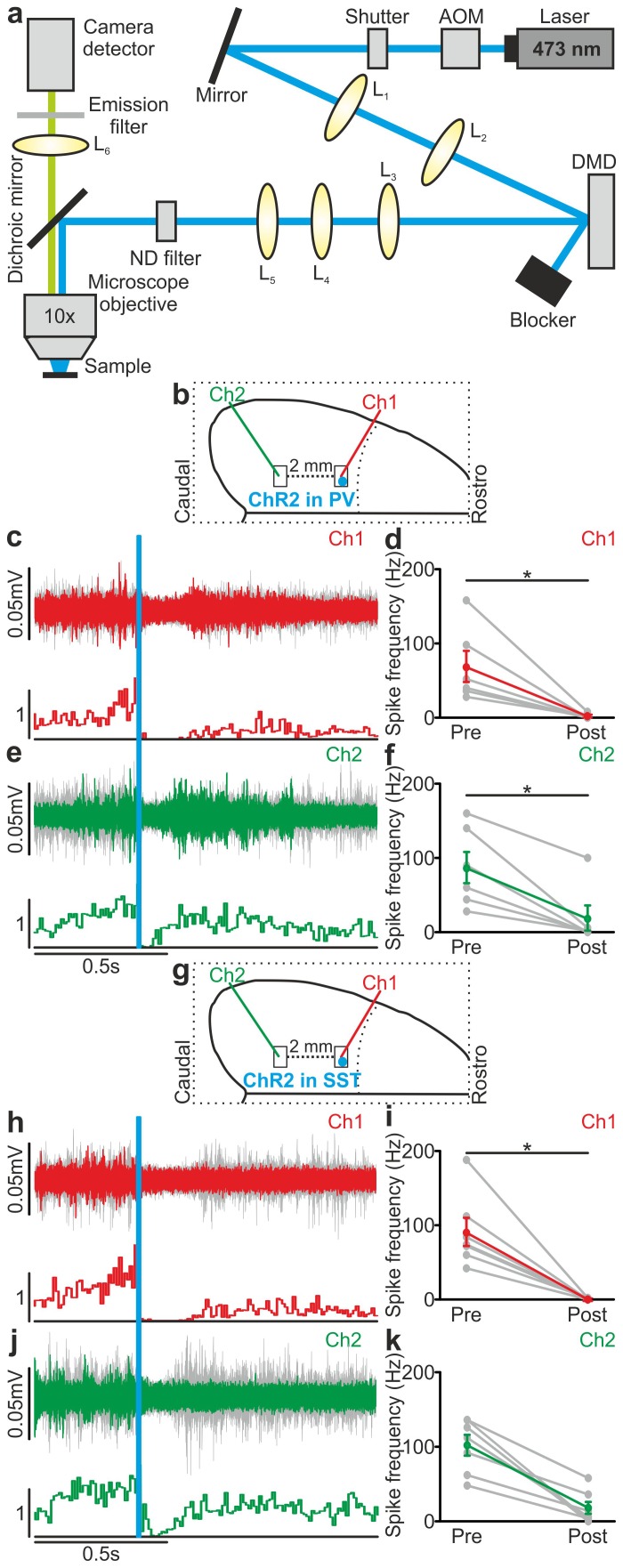
Large scale effect of PV and SST activation on multiunit activity using spatially-restricted DMD-based illumination. (**a**) Optical setup for patterned illumination with a DMD (see Materials and methods for details). (**b**) Schematic configuration for simultaneous extracellular recordings during photoactivation of PV interneurons expressing ChR2. The blue spot indicates the illuminated cortical region (200 µm diameter). (**c**) Top: example of multi-unit signals recorded in Ch1 during optogenetic activation of PV interneurons (blue line). Bottom: average PSTH of Ch1 for all recorded animals (N = 7 animals). (**d**) Average frequency of spikes recorded in Ch1 before (Pre) and after (Post) light stimulation (p = 3E-2, Wilcoxon signed-rank test, N = 6 animals). (**e**) Top: multi-unit traces in Ch2 simultaneously recorded with signals in a (top panel). Bottom: average PSTH of Ch2 for all recorded animals (N = 6 animals). (**f**) Average frequency of spikes recorded in Ch2 in the two time windows (p = 3E-2, N = 6 animals). (**g–k**) Same as in b-f but during optogenetic activation of SST interneurons expressing ChR2. In i, p = 2E-2, Wilcoxon signed-rank test, N = 7 animals. In k, p = 1E-3, paired Student’s *t*-test, N = 7 animals. **DOI:** http://dx.doi.org/10.7554/eLife.26177.048 10.7554/eLife.26177.049Figure 6—figure supplement 2—source data 1.Source data for the large scale effect of PV activation on multiunit activity using spatially-restricted DMD-based illumination.**DOI:** http://dx.doi.org/10.7554/eLife.26177.049 10.7554/eLife.26177.050Figure 6—figure supplement 2—source data 2.Source data for large scale effect of SST activation on multiunit activity using spatially-restricted DMD-based illumination.**DOI:** http://dx.doi.org/10.7554/eLife.26177.050

**Figure 6—figure supplement 3.**
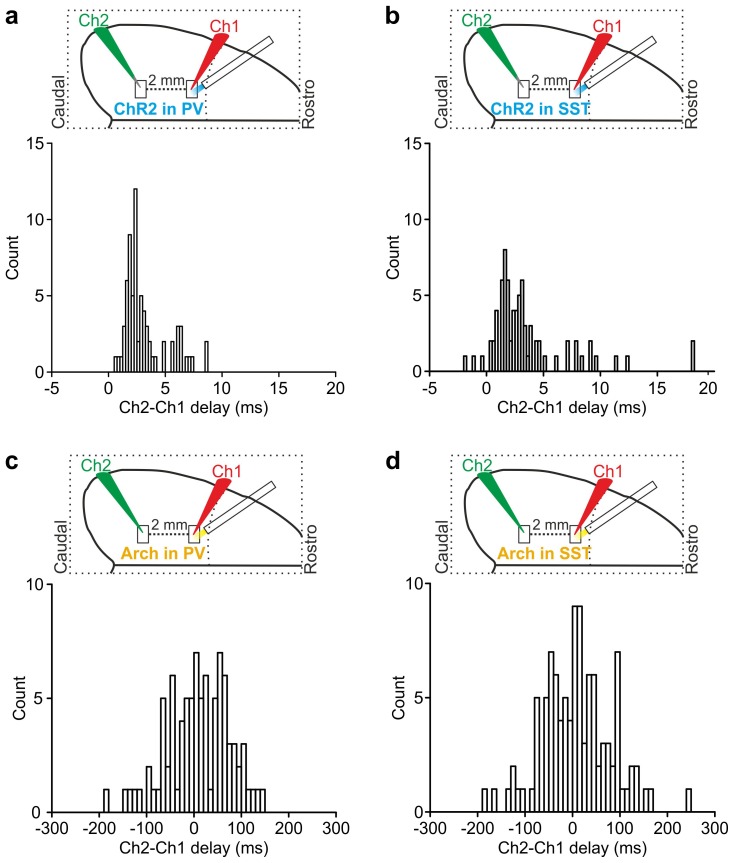
Temporal lag of optogenetically-induced state transitions across cortical areas. (**a**) Top: schematic of the experimental configuration for dual patch-clamp recordings from two cortical neurons (Ch1 and Ch2) located 2 mm apart during local optogenetic manipulation of PV cells in the area where Ch1 was recorded. Bottom: distribution of time lags (Ch2-Ch1 delay) of up-to-down transitions triggered by optogenetic activation of PV cells in the two simultaneously recorded neurons (bin width: 0.25 ms). (**b**) Same as in a for optogenetic activation of SST cells (bin width: 0.25 ms). (**c–d**) Same as in a-b for optogenetic inhibition of PV (**c**) or SST (**d**) neurons (bin width: 10 ms). **DOI:** http://dx.doi.org/10.7554/eLife.26177.051

**Figure 6—figure supplement 4.**
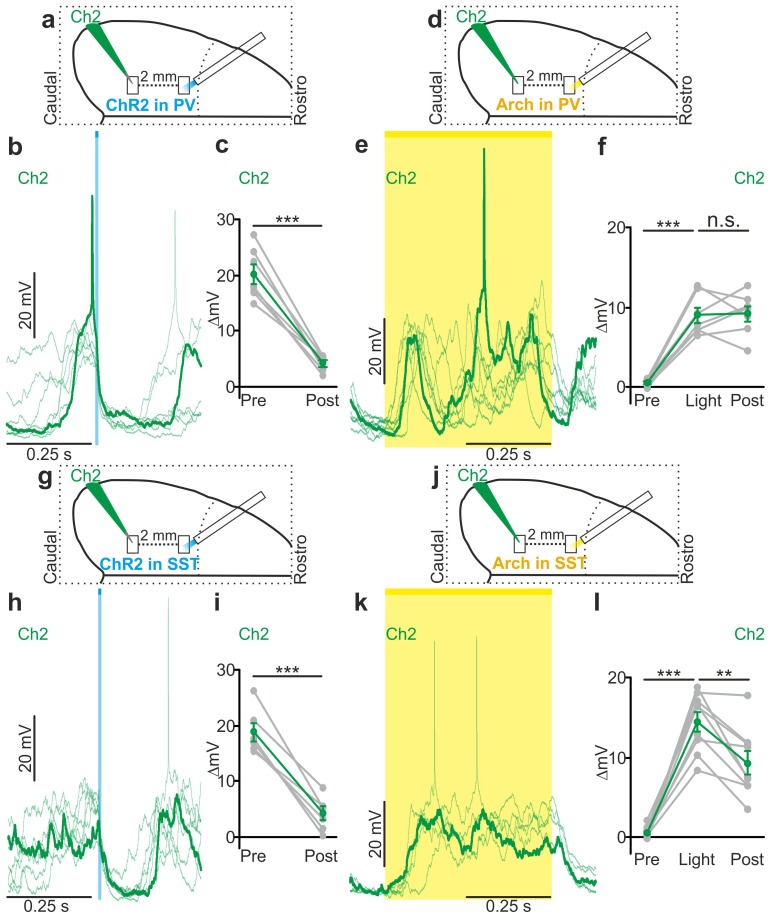
Local optogenetic modulation of interneurons modulates superficial pyramidal neurons over large cortical territories in non-anesthetized animals. (**a**) Schematic representation of the experimental configuration for intracellular recordings of superficial pyramidal neurons in non-anesthetized animals during photoactivation of PV interneurons expressing ChR2. Recorded neurons (Ch2, green) are located two millimetres apart from the illuminated region in the caudal direction. (**b**) Representative traces showing membrane potential effect of PV interneurons photoactivation (blue line) during up states. (**c**) Average membrane potential values before (Pre) and after (Post) light stimulation (p = 7E-5, paired Student’s *t*-test, N = 7 cells from three animals). (**d**) Schematic configuration for intracellular recordings of layer II/III pyramidal neurons during optogenetic inhibition of PV interneurons expressing Arch in non-anesthetized animals. Light stimulation is delivered two millimetres apart in the rostral direction from the recording site (Ch2, green). (**e**) Representative traces showing the effect of PV interneurons photoinhibition (yellow line) on the membrane potential of the recorded cell during down states. (**f**) Average membrane potential values before (Pre), during (Light) and after (Post) light stimulation (p = 1E-6, one-way ANOVA, N = 7 cells from four animals). (**g–l**) Same as in a-f but during optogenetic modulation of SST interneurons. In i, p = 3E-4, paired Student’s *t*-test, N = 6 cells from three animals. In l, p = 3E-4, one-way ANOVA, N = 9 cells from three animals. **DOI:** http://dx.doi.org/10.7554/eLife.26177.052 10.7554/eLife.26177.053Figure 6—figure supplement 4—source data 1.Source data for the analysis of membrane potential changes in pyramidal neurons located 2 mm far from modulated PV and SST cells in awake mice.**DOI:** http://dx.doi.org/10.7554/eLife.26177.053

**Figure 6—figure supplement 5.**
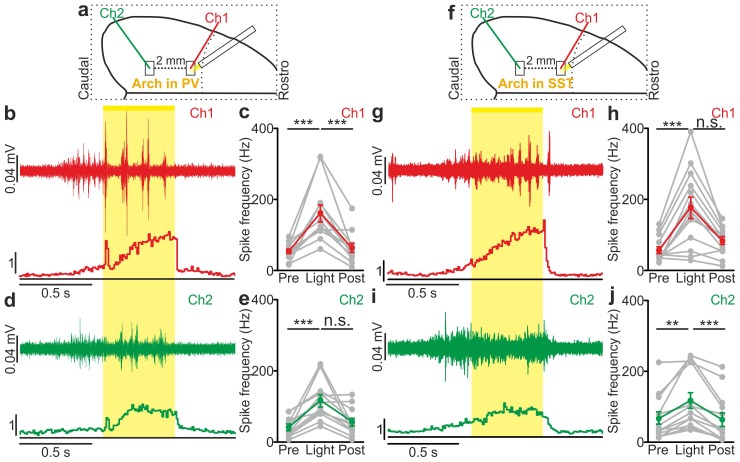
Local optogenetic inhibition of interneurons modulates network MUA over large cortical territories in anesthetized animals. (**a**) Schematic of the experimental setup. Simultaneous extracellular recordings are performed in anesthetized mice during photoinhibition of PV interneurons expressing Arch. (**b**) Top: example of a multi-unit signal recorded in Ch1 during optogenetic inhibition of PV interneurons (yellow line). Bottom: average PSTH of Ch1 for all recorded animals (N = 12). (**c**) Average frequency of spikes recorded in Ch1 before (Pre), during (Light) and after (Post) light stimulation (p = 1E-4, Friedman test, N = 12 animals). (**d**) Top: multi-unit signal recorded in Ch2 simultaneously with the signal in Ch1 shown in b (top panel). Bottom: average PSTH of Ch2 for all recorded animals (N = 12). (**e**) Same as in c but for recordings in Ch2 (p = 6E-4, Friedman test, N = 12 animals). (**f–j**) Same as in a-e but during photoinhibition of SST interneurons expressing Arch. In h, p = 2E-3, Friedman test, N = 13 animals. In j, p = 2E-4, Wilcoxon signed-rank test, N = 13 animals. **DOI:** http://dx.doi.org/10.7554/eLife.26177.054 10.7554/eLife.26177.055Figure 6—figure supplement 5—source data 1.Source data for the effect of local PV inhibition on network activity over large cortical territories.**DOI:** http://dx.doi.org/10.7554/eLife.26177.055 10.7554/eLife.26177.056Figure 6—figure supplement 5—source data 2.Source data for the effect of local SST inhibition on network activity over large cortical territories.**DOI:** http://dx.doi.org/10.7554/eLife.26177.056

**Figure 6—figure supplement 6.**
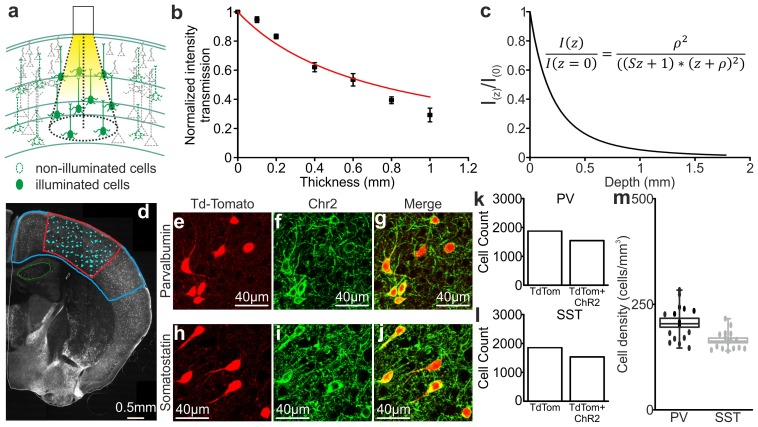
Light transmission through the cortical tissue and density of opsin positive cells. (**a**) Schematic representation of the illuminated brain volume using fiber optics. (**b**) Transmission fraction for yellow (λ = 594 nm) laser light as a function of the thickness of the cortical tissue. The red curve indicates the fit that was used to calculate the scattering coefficient (see Materials and Methods for details). (**c**) Light intensity as a function of cortical depth. The intensity values are normalized to the value at depth z = 0 mm. The inset shows the equation corresponding to the black curve. (**d**) An epifluorescence image of a representative coronal cortical section from a SST-Cre x TdTomato mouse injected with ChR2-eYFP that was used to evaluate cell density. The blue line indicates the region where the virus spread. The red line indicates the region with higher ChR2-eYFP expression, which was used for the cell count of TdTomato positive cells (light cyan dots indicate individual TdTomato positive cells). (**e–j**) Representative confocal images of PV (top) and SST (bottom) interneurons expressing TdTomato and ChR2 used to evaluate the percentage of double labelled (TdTomato and ChR2-eYFP) cells. (**k–l**) Total number of TdTomato positive (TdTom) or TdTomato and ChR2 positive (TdTom + ChR2) cells in mice expressing ChR2 in PV (shown in k) and SST (shown in l) interneurons. (**m**) A box plot of the density of double-labelled PV and SST cells in four different animals. The filled dots indicate the density values from all the individual coronal sections that were analysed. **DOI:** http://dx.doi.org/10.7554/eLife.26177.057 10.7554/eLife.26177.058Figure 6—figure supplement 6—source data 1.Source data for the evaluation of light transmission through cortical tissue and of the density of opsin-positive cells.**DOI:** http://dx.doi.org/10.7554/eLife.26177.058

## Discussion

During low-arousal states such as quiet wakefulness, NREM sleep, and some forms of anesthesia, cortical circuits display slow alternation of active (up) and silent (down) states (Steriade et al., 1993b, 1993a; Petersen et al., 2003a; Crochet and Petersen, 2006; Massimini et al., 2004). These activities play a fundamental role in shaping network function and in regulating fundamental cortical processes, including the modulation of sensory inputs (Petersen et al., 2003a; Haider et al., 2007; Reig et al., 2015), the regulation of synaptic plasticity (Rosanova and Ulrich, 2005; Vyazovskiy et al., 2008), and the control of memory consolidation (Diekelmann and Born, 2010; Miyamoto et al., 2016; Rasch et al., 2007). Given the relevance of up and down states in the control of cortical function, understanding the cellular mechanisms underlying the generation and modulation of these fundamental network dynamics is of crucial importance. Since the initial characterization of the up and down states of the slow oscillation using intracellular recordings (Steriade et al., 1993b), a role of interneurons in mediating the hyperpolarizing component of the up-to-down transitions was proposed. However, subsequent work has provided contradictory evidence. Electrophysiological recordings from individual interneurons showed that these cells fire during cortical up states (Steriade et al., 2001; Puig et al., 2008; Fanselow and Connors, 2010; Tahvildari et al., 2012; Neske et al., 2015) and perturbation of their activity during the up state modifies cellular dynamics (Neske and Connors, 2016), showing that interneurons are actively engaged during the up state. The timing of interneuronal spikes during the up state was shown to be variable (Puig et al., 2008) and dependent on the specific subtype of interneuron that was considered (Neske et al., 2015). Moreover, intracellular recordings in vitro and in vivo from principal cortical neurons showed paralleled changes in excitatory and inhibitory conductance during up state progression. Since no net increase nor gradual build-up of inhibitory conductance with respect to excitatory ones at the end of the up state was observed (Shu et al., 2003; Tahvildari et al., 2012; Neske et al., 2015), these data were taken against a role of interneurons in up state termination. Computational work also suggested that slow oscillatory activity and up state termination can occur in neural networks relying largely on adaptation currents in principal cells with a minor role of inhibition (Compte et al., 2003). However, pharmacological blockade of GABA_A_ receptors decreases the up state duration (Sanchez-Vives et al., 2010) and selectively antagonism of GABA_B_ receptors increases up state duration (Mann et al., 2009). Moreover, recent results pointed to a role of the GABA_A_ receptor in the synchronous termination of the up state across different cortical areas (Lemieux et al., 2015), suggesting that interneurons may contribute to terminate up states as also supported by other in silico work (Bazhenov et al., 2002; Chen et al., 2012).

### Interneurons control the up-to-down transition

In the present study we demonstrate that activity of the two major subtypes of cortical interneurons, the PV and the SST positive cells, causally contribute to terminate cortical up states. Their causal involvement was supported by two independent experimental approaches: (i) in non-perturbative experiments in which we measured the spike activity of identified interneuronal subtypes using two-photon guided juxtasomal electrophysiological recordings while measuring network dynamics with the LFP; (ii) using bidirectional optogenetic perturbations of GABAergic cells to modulate their firing activity while recording network and single cell activity with electrophysiology. In the first approach, we found that interneuronal spikes are more strongly locked to future than to past variations in the LFP phase, and that interneuronal spiking during up states was associated with subsequent acceleration of the LFP phase speed (Figure 2). In the second approach, we found that brief optogenetic activation of either PV or SST interneurons resulted in the reduction of network activities in extracellular LFP and MUA recordings (Figure 3—figure supplements 4–5). Moreover, in intracellular patch-clamp experiments from principal neurons we observed membrane hyperpolarization resembling a full transition to the down state following PV or SST optogenetic activation (Figure 3). Optogenetic inhibition of PV and SST interneurons during an ongoing up state prolonged the up state for the entire duration of the light stimulus (Figure 4), further supporting that interneuronal firing contributes to up state termination. Our conclusions were strengthened by finding compatible results for the causal role of these interneurons in slow oscillation dynamics both with statistical analysis of fully unperturbed endogenous neural activity (Figure 2) and with optogenetic manipulations (Figures 3–4). These results demonstrate the value of optimally combining optogenetics with statistical analysis of neural activity recorded under unperturbed natural conditions to dissect the causal role of specific circuit elements in network dynamics (Panzeri et al., 2017).

These results do not contrast with previous studies reporting similar dynamics of inhibitory conductances with respect to excitatory ones at the end of the up state (Shu et al., 2003; Haider et al., 2007; Neske et al., 2015). First, the lack of a net increase of inhibition with respect to excitation at the end of the up state does not necessarily exclude a causal role of interneurons in promoting up-to-down transition. Second, conductance measurements that are typically averaged across oscillation cycles may have hindered variable features of inhibitory networks associated to the stochastic nature of cortical dynamics, as for example transient changes in interneuronal synchrony. It may end up that up-to-down state transitions are associated with lower but more synchronous firing activity in subsets of interneurons innervating specific compartments of principal neurons. To test this hypothesis further experiments encompassing simultaneous LFP recordings and two-photon calcium imaging from different subtypes of interneurons or multiple juxtasomal recordings from identified GABAergic cells will be needed.

### Interneurons control the down-to-up transition

Remarkably, our data also demonstrate a previously unrecognized role of interneurons in the control of down state duration. Photo-inhibition of PV or SST interneurons during ongoing down states triggered reliable transitions to active cortical states. These transitions resembled spontaneous up states as they were characterized by membrane depolarization in intracellular recordings (Figure 4), an increase in gamma frequencies in the LFP and an augmentation of MUA signals at the network level (Figure 4—figure supplements 3–4). These results were surprising given that interneurons were commonly believed to be largely inactive during cortical down states (Timofeev et al., 2001; Tahvildari et al., 2012) (but see also [Fanselow and Connors, 2010; Neske et al., 2015; Ushimaru and Kawaguchi, 2015]). However, our juxtasomal electrophysiological recordings in vivo showed that a small but significant fraction of interneuronal spikes also occurs in down states. Based on these results, we propose that the weak firing activity of interneurons during cortical down state is fundamental to maintain the cortical circuitry in the silent state, preventing it from escaping into the up state. Chloride imaging across different compartments of pyramidal neurons during up and down state transitions might be used as an alternative experimental approach to further investigate this issue.

In this context it is interesting to evaluate how many spikes interneurons need to discharge to maintain a cortical down state and how many spikes the optogenetic manipulations used in this study are interfering with. To address these questions, we first estimated the number of illuminated cells that we efficiently silenced during optogenetic inhibitory experiments by calculating the volume of the illuminated brain region in which interneurons were reliably modulated by light and the density of opsin positive cells under our experimental conditions (Figure 6—figure supplement 6d–m, see Materials and methods for details). Based on our calculations, we estimated that ~100 PV cells and ~650 SST neurons were efficiently modulated during our optogenetic manipulations. From the data about spike activity of interneurons during down states reported in Figure 1, we then calculated that the optogenetic inhibitory manipulations displayed in Figure 4 interfered with ~40 APs in PV cells and about ~20 APs in SST interneurons during down states. These results suggest that the GABAergic control of cortical state transition is surprisingly sensitive, with only a few tens of APs fired by interneurons efficiently controlling down-to-up state transition.

### Inhibitory modulation of cortical state transition is interneuron-subtype specific

The network effects of optogenetic manipulation of PV and SST interneurons were interneuronal type specific. PV cells exerted a stronger effect on state transitions compared with SST interneurons (Figure 5), but manipulation of SST cells triggered state transitions that more closely resembled spontaneous transitions compared to manipulation of PV interneurons. During both up and down states, photo-inhibition of PV, but not SST interneurons, significantly increased the spiking activity of pyramidal neurons. Moreover, PV photo-inhibition during down states increased the probability of generating down-to-up transitions with shorter latencies and smaller temporal variance compared to photo-inhibition of SST cells. Finally, optogenetic inhibition of SST, but not PV interneurons, triggered down-to-up state transitions with slope similar to spontaneous transitions. These differences in modulating the activity of pyramidal neurons can be explained based on at least three different properties of PV and SST cells: (i) the average firing rate; (ii) the location of synaptic contacts onto principal cells; (iii) the strength of the synaptic connections. For example, the stronger effect of PV photo-inhibition on network activities might be related to the higher firing activity of PV cells compared to SST interneurons (Gentet et al., 2010; Gentet, 2012; De Stasi et al., 2016). In line with this interpretation, the higher firing rate of SST cells in awake compared to anesthetized animals (Vyazovskiy et al., 2008) may also explain the stronger effect of SST inhibition observed in awake compared to the anesthetized condition (Figure 5).

Although the phase locking (Figure 2b,h) and phase speed analysis (Figure 2f,l) on simultaneous LFP and juxtasomal recordings do not show major differences between PV and SST cells, optogenetic inhibitory manipulation of SST interneurons triggered cortical state transitions that better resembled spontaneous transition compared to the same manipulation performed on PV cells. These latter results could point to a more prominent role of SST cells in regulating cortical state dynamics under physiological conditions. This would be in line with recent findings demonstrating that SST firing activity is extremely sensitive to cortical states and strongly affected by cholinergic inputs from subcortical areas (Gentet et al., 2010; Chen et al., 2015; Muñoz et al., 2017).

It is worth noting that the lack of significant difference between the effect on up-to-down state transition of optogenetic activation of PV and SST interneurons (Figure 3) might be due to the oversynchronous activity induced in interneurons by the brief light pulse that we used in the current study. A ramp-like light stimulus (Adesnik and Scanziani, 2010) or stabilized step function opsins (Berndt et al., 2009) coupled with minimal light intensity stimulation could be used to elevate interneuronal excitability without explicitly controlling spike timing across the population. With this approach, it might be easier to unmask differences between the various interneuron subtypes because any sort of synchronous spiking would emerge from the natural statistics of the spike trains of the population under study rather than be imposed by the optogenetic manipulation.

### Potential mechanisms driving interneuronal firing during down states

Local excitatory connectivity between principal cells and interneurons contribute to drive the activity of cortical inhibitory cells during the ongoing network dynamics (Dantzker and Callaway, 2000; Staiger et al., 2009; Apicella et al., 2012). An unanswered question is then what drives interneurons to fire during cortical down states, when excitatory cells have been reported to be largely silent. One possibility is that GABAergic cells undergo intrinsic oscillatory activity as for example reported in (Le Bon-Jego and Yuste, 2007) or that neuromodulators may regulate the excitability of interneurons (Chen et al., 2015). Alternatively, small subpopulations of excitatory neurons were reported to fire during cortical down states (Neske et al., 2015; Ushimaru and Kawaguchi, 2015) and this mechanism may drive certain interneurons to fire. A third possibility is that long-range excitatory fibers from other brain regions may input onto local cortical interneurons. In this regard, it is interesting to note that spiking activity of thalamic nuclei have been reported to occur during cortical down states ahead of up state initiation and it has been proposed that the early activation of thalamic nuclei contributes to the generation of up states by early activation of fast spiking (FS) cells followed stimulation of pyramidal neurons (Ushimaru and Kawaguchi, 2015). Our data are compatible with these experimental findings, but suggest that early activation of FS interneurons may actually prevent, rather than favour, the up state initiation. While it is clear that *strong* thalamic activation by means of electrical (MacLean et al., 2005; Reig and Sanchez-Vives, 2007; Reig et al., 2015; Watson et al., 2008) or sensory (Petersen et al., 2003a; Hasenstaub et al., 2007; Civillico and Contreras, 2012) stimulation is a trigger for down-to-up state transitions in cortex, our data are compatible with the scenario in which *weak* thalamic activation during cortical down states preferentially induces suprathreshold activity in cortical FS interneurons (Hu and Agmon, 2016) leading to a prolongation of the silent cortical state. In this framework, the observation that SST interneurons are also active during cortical down states suggests that these interneuronal cells may be similarly controlled by thalamic inputs (Tan et al., 2008).

### Local modulation of inhibition controls network states over large cortical territories

Using simultaneous recordings of network and single cell activity in two brain regions located ~2 mm apart, we demonstrated that local activation of interneurons in one of the two regions during an up state causes a transition to the down state that occurs near synchronously in both recorded areas (Figure 6 and Figure 6—figure supplement 1). These results were not due to unwanted direct illumination of both recorded cortical areas because we replicated these findings both with local illumination through a fiber optic and with patterned illumination that precisely delivered light to one restricted region of interest in the sample (Figure 6—figure supplement 2). Previous reports showed that up-to-down transitions occur with short (i.e. few milliseconds) delay among cortical regions (Volgushev et al., 2006; Chen et al., 2012), but the mechanisms underlying this phenomenon are not understood. Our data show that photoactivation of interneurons in one cortical region promoted up-to-down transitions over large territories (up to 2 mm apart from the illuminated site) with latencies as short (~3 ms) as the ones characterizing spontaneous up-to-down transitions (Volgushev et al., 2006; Chen et al., 2012). This suggests that a sudden withdrawal of activity in a confined cortical area can trigger synchronous mesoscale network transitions towards silent states. Moreover, our data also show that photo-inhibition of interneurons during an ongoing up state significantly affects network activity at distal cortical locations. When interneurons were locally inhibited in one region during a down state a transition to the up state was reliably observed in two recorded areas located >2 mm apart (Figure 6e,f,k,l), directly demonstrating a role of local inhibition in the control of down state synchrony across cortical areas. This observation is also of crucial importance for the correct interpretation of past works, where local optogenetic perturbation of interneurons was used to silence activity and this manipulation was assumed to have an effect mostly in the proximity of the illuminated area (Adesnik et al., 2012; Lee et al., 2012; Gentet, 2012; Sachidhanandam et al., 2013).

In conclusion, our data provide important, new and quantitative insights in the role of distinct inhibitory sub-networks in the control of cortical spontaneous dynamics. We show that the discharge of a small number (few tens) of APs in specific classes of local interneurons controls mesoscale state transitions in cortex. Because network state is known to powerfully and dynamically modulate several cortical functions, including sensory processing (Petersen et al., 2003a; Haider et al., 2007; Reig and Sanchez-Vives, 2007), our findings might have important implications for the understanding of the cellular mechanisms underlying the dynamics of flexible computational processes in the cortex.

## Materials and methods

### Animals

Experimental procedures involving animals have been approved by the IIT Animal Welfare Body and by the Italian Ministry of Health (authorization # 34/2015-PR and 125/2012-B), in accordance with the National legislation (D.Lgs. 26/2014) and the European legislation (European Directive 2010/63/EU). The mouse lines B6;129S6-*Gt(ROSA)26Sor^tm14(CAG-TdTomato)Hze^/J*, id #007908, RRID:IMSR_JAX:007908 (otherwise called TdTomato line), *Pvalb^Cre^*, B6.129P2-*Pvalb^tm1(cre)Arbr^/J*, id #017320, RRID:IMSR_JAX:017320 (called PV-cre line) and *Sst^Cre^*, *Sst^tm2.1(cre)Zjh^/J*, id #013044, RRID:IMSR_JAX:013044 (called SST-cre line) were purchased from the Jackson Laboratory (Bar Harbor, USA). The animals were housed in a 12:12 hr light-dark cycle in singularly ventilated cages, with access to food and water *ad libitum*.

### Viral injections

The adeno-associated viruses (AAVs) AAV2.1.EF1a.DIO.hChR2(H134R)-EYFP.WPRE.hGH, AAV2.1EF1.dflox.hChR2(H134R)-mcherry.WPRE.hGH, AAV2.1.flex.CBA.Arch-GFP.WPRE.SV40, and AAV2.9.flex.CBA.Arch-GFP.WPRE.SV40 were purchased from the University of Pennsylvania Viral Vector Core. PV-Cre, SST-Cre, PV*x*TdTomato, and SST*x*TdTomato transgenic mice (both males and females) were injected between postnatal day 0 (P0) and P2. Pups were anesthetized using hypothermia, placed on a custom-made stereotaxic apparatus and kept at approximately 4°C for the entire duration of the surgery. The skull was exposed through a small skin incision and ~250 nl of viral suspension were slowly injected using a glass micropipette at stereotaxic coordinates of 0 mm from bregma, 1.5 mm lateral of the sagittal sinus and 0.25–0.3 mm depth. Following injection the micropipette was held in place for 1–2 min before retraction. After pipette removal, the skin was sutured and the pup was revitalized under an infrared heating lamp.

### Procedure for in vivo recordings

Electrophysiological experiments were performed at postnatal day P24-P28 for PV-Cre mice and P24-P30 for SST-Cre animals. For experiments performed in anesthetized animals, mice were injected intraperitoneally with urethane (16.5%, 1.65 g/kg). The body temperature was monitored using a rectal probe and maintained at 37°C with a heating pad. Oxygen saturation was controlled by a pulse oximeter (MouseOx, Starr Life Sciences Corp., Oakmont, PA). Respiration rate, heartbeat, eyelid reflex, vibrissae movements, reactions to tail and toe pinching were monitored to control the depth of anaesthesia throughout the surgery and the experiment. At the beginning of surgical procedure, 2% of lidocaine solution was subcutaneously applied in the proximity of the site of craniotomy. The position of the craniotomy was generally guided by the maximal intensity of the fluorescence. In many experiments, this coincided with the somatosensory area of the neocortex. Once the craniotomy was opened, the brain surface was kept moist with a HEPES-buffered artificial cerebrospinal fluid (ACSF). The dura was removed with a metal needle only for extracellular electrophysiological recordings. For simultaneous juxtasomal and Local Field Potential (LFP) recordings (Figures 1 and 2, Figure 1—figure supplement 1 and Figure 2—figure supplements 1–4) two different craniotomies (<0.5×0.5 mm^2^) were performed at 0.5 mm distance one from the other, while for dual patch-clamp and extracellular recordings the distance between the two craniotomies was ~2 mm (Figure 6, and Figure 6—figure supplements 1–5).

For in vivo experiments in non-anesthetized head-restrained animals, 2 weeks before the recording session mice were anesthetized with isofluorane 2.5% and a custom metal plate was fixed with dental cement on the skull. After a 2–3 days recovery period, animals were habituated to sit quietly on the experimental rig while their head was fixed. Habituation was performed for a minimum of 7–10 days. One training session per day was performed and the duration of the training session gradually increased each day (from 15 min to 1 hr). The day of the experiment, mice were anesthetized with isofluorane 2.5% and a craniotomy was opened on the targeted area as described above. After the surgery, mice recovered for at least 30 min before the beginning of the experimental session.

### Simultaneous LFP and two-photon-guided juxtasomal recordings in vivo

Double transgenic PV-Cre *x* TdTomato and SST-Cre *x* TdTomato mice were used for these experiments. A low resistance (0.3–0.6 MΩ) pipette was filled with ACSF, lowered in the craniotomy at ~300 µm depth from the pial surface and used to monitor the superficial LFP activity. A second glass pipette with higher resistance (5–8 MΩ) filled with ACSF and 2 mM Alexa 488 Fluor (Thermo Fisher Scientific, Waltham, MA, USA) was used for juxtasomal electrophysiological recordings. The second pipette was placed 80–350 µm below the pial surface and PV or SST positive interneurons were identified by imaging TdTomato fluorescence with an Ultima II laser scanning two-photon microscope (Bruker Corp., Billerica, MA, former Prairie Technologies, Madison, WI, USA) coupled to a Chameleon Ultra II (Coherent Corp., Santa Clara, CA, λ_exc_ = 720 nm). When the tip of the pipette and the targeted cell were in close contact, a negative pressure was applied to the pipette in order to achieve the juxtasomal recording configuration (resistance >20 MΩ). For LFP recordings, the electrical signal was amplified using an AM-amplifier (AM-system, Carlsborg, WA, USA), digitized at 10 kHz and stored with PatchMaster software (RRID:SCR_000034). Spiking activity from juxtasomal recordings was acquired with an ELC-01X Amplifier, digitized (10 kHz) and stored with the same software as for the LFP signal.

### Extracellular and intracellular recordings in vivo

LFP and multi-unit activity (MUA) were acquired using custom-built probes made of two parallel tungsten electrodes (FHC Inc., Bowdoin, ME, USA). The distance between the tips of the electrodes was ~200–250 µm. Electrodes were lowered into the tissue with the deeper tip placed at ~350 µm from pial surface. For simultaneous recordings of MUA (Figure 6—figure supplements 1, 2 and 5) in two cortical regions, two different probes were inserted in the same hemisphere 1.5–2 mm away from each other in the rostro-caudal direction and lowered at the same cortical depth. Electrical signals were filtered at 0.1 Hz–5 kHz, amplified by an AM-amplifier (AM-system, Carlsborg, WA, USA) and digitized at 50 kHz with a Digidata 1440 (Axon Instruments, Union City, CA).

Current-clamp patch-clamp recordings were carried out on superficial pyramidal neurons (100–350 μm). 3–6 MΩ borosilicate glass pipettes (Hilgenberg, Malsfeld, Germany) were filled with an internal solution containing (in mM): K-gluconate 140, MgCl_2_ 1, NaCl 8, Na_2_ATP 2, Na_3_GTP 0.5, HEPES 10, Tris-phosphocreatine 10 to pH 7.2 with KOH. In some experiments byocitin (3 mg/ml) was also added for post hoc cell identification and reconstruction. Electrical signals were acquired using a Multiclamp 700B amplifier, filtered at 10 kHz, digitized at 50 kHz with a Digidata 1440 and stored with pClamp 10 (RRID:SCR_011323, Axon Instruments, Union City, CA).

### Optical stimulation

Continuous wave, solid-state laser sources (CNI, Changchun, China; World Star Tech, Toronto, Canada; Cobolt, Vretenvägen, Sweden) were used to deliver blue (λ = 473 nm, 488 nm or 491 nm, stimulus duration 10 ms, unless otherwise stated) or yellow (λ = 594 nm, stimulus duration 500 ms) light illumination through an optical fiber (fiber diameter: 200 µm; fiber numerical aperture: 0.22; AMS Technologies, Milan, Italy) or, for patterned illumination experiments, via a 10X object (Olympus, Tokyo, JP), coupled to a digital mirror device (DMD). Light power used for blue light illumination ranged between 0.2–18 mW, while for yellow light between 0.12–30 mW. Laser power was measured at the fiber tip or underneath the objective. During in vivo recordings, the optical fiber was placed in close proximity to the pial surface above the recording site.

For patterned illumination (Figure 6—figure supplement 2), the beam was expanded by a first telescope using achromatic doublet lenses (L_1_ and L_2_ in Figure 6—figure supplement 2a; respectively AC254-035-A and AC254-150-A, Thorlabs, Dachau, DE) to impinge on the active window of the DMD (V-7000 module, ViALUX Chemnitz, DE) with an angle of −24° with respect to the direction normal to the DMD active window. The ON axis component of the modulated beam (exiting at 0° with respect to the direction normal to the DMD active window) was then relayed by a series of doublets lenses (L_3_, L_4_, L_5_ in Figure 6—figure supplement 2a, respectively AC254-100-A, AC254-060-A and AC254-100-A, Thorlabs) and a 10X microscope objective (UPlanFLN 10 × 0.3 NA, Olympus, Tokyo, JP) to the sample. Laser intensity was modulated by an acousto-optic modulator (AOM, R23080-3-LTD, Gooch and Housego, Ilminster, UK) and neutral density filters (NEK01, Thorlabs) positioned at the beam exit from the laser for experimental purposes. Before entering the microscope objective the beam went through a dichroic mirror (Di01-R404/488/594, Semrock, Rochester, NY, USA). Fluorescence emission was collected through a lens (L_6_, f = 180 mm, U-TLUIR, Olympus, Tokyo, JP) by a camera (ORCA-Flash4.0, Hamamatsu, Hamamatsu, JP) with an appropriate emission filter in front of it. The DMD was controlled using custom-made software written in LabVIEW (RRID:SCR_014325, National Instruments, Austin, TX), which manage the communication with the ViALUX driving board using the ALP-4.1 controller suite. The ALP-4.1 Application Programming Interface (API) allowed loading the patterns to an on-board memory, setting triggers and stimulation time, and managing other driver functionalities. Calibration was performed projecting a rectangular pattern, adapting it to the pre-calibrated camera field of view, and retrieving the mapping parameters between DMD and sample plane. In experiments displayed in Figure 6—figure supplement 2, a circular shape (diameter: 200 µm) was projected onto the surface of the brain close to one of the two recording electrodes (Ch1).

To measure the fraction of light transmitted through the brain tissue, the same optical fiber used for in vivo experiments was placed perpendicularly to the slice (slice thickness: 0.05–1 mm) and in close proximity to the tissue surface. Transmitted laser power was measured by placing the sensor of the power meter underneath the cortical slice. The ‘transmission fraction’ was calculated as the ratio between the measured laser power in the presence of the cortical tissue and the maximal laser power obtained in absence of the cortical slice (Figure 6—figure supplement 6b). The transmission fraction was plotted as a function of the slice thickness and the scattering coefficient was obtained from data interpolation as in Aravanis et al. (2007).

The volume of tissue in which cells where efficiently modulated by light (0.0193 mm^3^ for PV cells and 0.159 mm^3^ for SST interneurons) was calculated based on the diameter of the optical fiber (200 µm), the numerical aperture of the optical fiber (0.22), and the maximal depth at which interneurons could be reliably modulated by light (320 µm for PV cells and 1 mm for SST interneurons). The latter parameter was calculated from the equation displayed in Figure 6—figure supplement 6c. The minimal laser power (I_z_) required to efficiently silence interneurons was evaluated in in vitro recordings in coronal slices and corresponded to the laser power required to completely silence interneurons during a step of current injection (150–750 pA) that elicited sustained firing activity. I_z_ was ~1.4 mW and ~0.5 mW for PV and SST cells, respectively. For PV interneurons, I_z = 0_ (7.5 mW) was calculated as the minimal laser power value that, in inhibitory optogenetic experiments in vivo, prolonged the up state, increased up state generation probability, and increased spiking activity during up and down states. For SST cells, I_z = 0_ (16 mW) was calculated based only on the first two parameters due to the absence increased spiking activity in pyramidal cells after photoinhibition of SST interneurons.

### Slice electrophysiology

Cortical slice were prepared as described previously (Beltramo et al., 2013). For patch-clamp recordings pipettes (tip resistance, 3–4 MΩ) were filled with (in mM): K-gluconate 140, MgCl_2_ 1, NaCl 8, Na_2_ATP 2, Na_3_GTP 0.5, HEPES 10, Tris-phosphocreatine 10 to pH 7.2 with KOH. Extracellular solution was: 125 NaCl, 2.5 KCl, 1.25 NaH_2_PO_4_, 25 NaHCO_3_, 2 MgCl_2_, 2 CaCl_2_, 25 glucose, pH 7.4 with 95%O_2_/5% CO_2._ Series resistance (range 6–20 MΩ) was not compensated and data were not corrected for the liquid junction potential. The system for signal amplification, digitalization and storage (Axon Instruments, Union City, CA) was the same used for intracellular recordings in vivo (see above). For voltage-clamp experiments, the signal was sampled at 10 kHz and filtered at 2 kHz.

### Immunohistochemistry, cell morphology reconstruction, and cell density measurement

For immunofluorescence analysis, animals were deeply anesthetized with urethane (16,5%) and transcardially perfused with 0.01 M PBS (pH 7.4) and 4% paraformaldehyde in PBS. Brains were post-fixed overnight and cryoprotected with 30% sucrose in PBS. Coronal slices (40 µm-thick) were then cut and sequentially collected. Slices were incubated for 48 hr at 4°C with primary antibody diluted in PBS containing 5% normal serum of the same species as the secondary antibody, 0.3% Triton-X 100% and 0.01% sodium azide, and then placed for 2–3 hr at room temperature (RT) with the appropriate secondary antibody. Counterstain with Hoechst (1:400, 30 min RT) was performed before slices were mounted on glass slides with 1,4diazobiocyclo-(2,2,2)octane (DABCO)-based antifade mounting medium and finally coverslipped. The following antibodies were used for the immunofluorescence procedures: anti-GABA (RRID:AB_477652, 1:100 rabbit, Sigma A2052); anti-parvalbumin (RRID:AB_477329, 1:1000 mouse, Sigma P3088); anti-somatostatin (RRID:AB_2255365, 1:200 rat, Millipore MAB 354). Secondary antibodies consisted of: goat anti-rabbit 488 (RRID:AB_2576217, 1:800, Invitrogen A11034), goat anti-rabbit 647 (RRID:AB_2535813, 1:800, Invitrogen A21245), goat anti-mouse 488 (RRID:AB_2534088, 1:800, Invitrogen A11029); goat anti-rat 647 (RRID:AB_141778, 1:800, Invitrogen A21247). Confocal high-resolution images (2048 × 2048 pixels; Leica SP5, Wetzlar, DE) were used for cell count analysis displayed in Figure 3—figure supplement 1. Slices were randomly chosen in a rough volume (~1.5 mm radius) around the injection site and cell count was restricted to the supragranular layers of the cortex.

Biocytin-filled neurons were stained using the following protocol: coronal slices (250 μm) were incubated for 20 min in 3% H_2_O_2_ containing PBS solution for peroxidase inactivation, permeabilized for 1 hr RT with 2% Triton X-100 solution and subsequently kept overnight at 4°C with the avidin-biotin HRP complex (ABC, Vector Laboratories, Burlingame, CA, USA) solution containing 1% Triton X-100. The day after, slices were washed with PBS and then incubated with DAB (DAB Peroxidase Substrate Kit, 3, 3’-diaminobenzidine, Vector Laboratories, Burlingame, CA, USA). The reaction was monitored under a stereomicroscope and stopped when labelled neurons became visible. Slices were finally mounted with DABCO. Morphological reconstruction was performed with Neurolucida (RRID:SCR_001775, MicroBrightField Williston, VT, USA).

To calculate opsin-positive cell density, PV-Cre or SST-Cre x TdTomato mice were injected with ChR2. The cortical region was selected following ChR2 expression and TdTomato-positive cells were counted using a systematic random sampling method applied to four consecutive sections per animal. Sampling was performed by applying a virtual counting grid (square’s size 80 × 80 µm) over the whole area of interest using Neurolucida (Micro-BrightField, Colchester, VT, USA). Cells were counted throughout the whole thickness of the slice (40 µm) counting sequentially in one out of each four squares of the grid. Cells contacting a line on the upper or left edge of the grid element were excluded and cells contacting the lower or right edge of the grid element were included in the count. The number and position of each cell in the counted area were marked. Cell planar density (number of labelled cells / mm^2^) was calculated, and the total number (T) of labelled cells within a given volume V was estimated as: T = (N x V) / t, where N is the cell density and t the thickness of the sections. The fraction of TdTomato-positive neurons which was also positive for ChR2 (ChR2-eYFP/Tdtomato double-labelled cells) was evaluated on confocal z-stacks (1 µm steps; Leica SP5, Wetzlar, DE) on the same sections and same cortical areas used for the measurement of the density of TdTomato-positive cells. The percentage of double-labelled cells was used to finally estimate the total number of opsin-expressing cells within the illuminated volume.

### Up and down state detection from LFP signal

The raw LFP signal was first low-pass filtered below 500 Hz (second-order elliptic filter, 0.1 dB peak-to-peak passband ripple, 40 dB stopband attenuation down from the peak passband value) and then down-sampled to 1 kHz. Up and down states were then detected from the so-processed LFP using a method based on combining the approach of Saleem and colleagues (Saleem et al., 2010) (based on the instantaneous phase of the LFP in the low-frequency <4 Hz band), with a modified version of the algorithm proposed by Mukovski et al. (2007) (exploiting differences in beta and gamma-band power between up and down states). Since the Saleem method depended on the choice of two low-frequency bands, whose instantaneous phase was used for state detection, as well as on a few angular parameters that may vary according to the specific recording configuration, we optimized these parameters for our experimental conditions by using six simultaneous LFP and patch-clamp recordings on pyramidal neurons (Figure 1—figure supplement 1). The MATLAB source code used to perform these calculations is available as a supplementary material file (Source code 1). Up/down states were first detected from membrane potential traces of pyramidal neurons (Figure 1—figure supplement 1a) and those results were used as ground-truth data to calibrate and estimate the performance of the up/down state detection from the LFP signal (Figure 1—figure supplement 1b). To detect up/down states, membrane potential traces were analysed as proposed by (Saleem et al., 2010), with the only difference that we set the minimum interval between consecutive states at 50 ms. To facilitate the comparison with the method of (Saleem et al., 2010) we set the polarity of the LFP so that the ‘troughs’ of the LFP corresponded to up states and the peaks of the LFP to down states. We found that under our conditions the optimal bands for state detection were [0–1 Hz] and [1–3 Hz], slightly different values with respect to the optimal bands [0–2 Hz] and [2–4 Hz] (Figure 1—figure supplement 1f) found in Saleem et al. (2010). Moreover, to assess the possible effect of the method used to extract the instantaneous LFP phase, we computed the phase either as the angle of the Hilbert transform or by using linear interpolation between peaks/troughs and zero-crossing points (Eschenko et al., 2012). We found that the phase computed by Hilbert transform gave better state detection performance, thus we reported only results referring to states detected using Hilbert phase (Figure 1—figure supplement 1g).

The output of the Saleem method is a decision (or evidence) variable *S_delta_(t)*, computed by combining the differential likelihood of observing an up or down state from the chosen bands, which varies between 0 and 1 and can be used to determine the instantaneous state. To also take advantage of the information about the state given by higher frequencies, following (Mukovski et al., 2007) we combined *S_delta_(t)* with another decision variable extracted from the LFP in the 10–40 Hz range including the beta and gamma bands (*S_beta−gamma_(t)*). We chose to use such band because we noticed that in this frequency interval there are the highest differences between power spectra as computed during either up or down states (Figure 1—figure supplement 1d). To calculate *S_beta−gamma_(t)*, we first processed the filtered signal to accentuate the difference between the periods of high-amplitude fluctuations and those of low amplitudes in the [10, 40] Hz band. To do that, we calculated the standard deviation (corresponding to the root mean square) of the filtered signal in a running frame of 5 ms. Then, we smoothed the obtained trace with a 50 ms running frame linear filter (Mukovski et al., 2007). Finally, the resulting signal has been normalized between 0 and 1, in order to be averaged with *S_delta_(t)* and obtain *S_comb_(t)* (Figure 1—figure supplement 1c). We show in Figure 1—figure supplement 1e that the performances obtained by the algorithm when considering *S_comb_(t)* are slightly higher than when using *S_delta_(t)* alone; hence, we decided to use *S_comb_(t)* instead of *S_delta_(t)* to estimate the state at each time instant.

To determine the thresholds for the detection of up/down states, the distribution of *S_comb_(t)* was fitted by a mixture of three Gaussians using an expectation maximization algorithm (Saleem et al., 2010). Each Gaussian represents a different cortical state, that is, up (highest values of *S_comb_*), down (lowest), and indeterminate (intermediate). Time samples corresponding to *S_comb_(t) > μ_UP_ − 2σ_UP_* were assigned to up states, and samples corresponding to *S_comb_(t) > μ_DOWN_ + 2σ_DOWN_* to down states (where means and variances of the Gaussians are represented as *μ_UP_, μ_DOWN_*, and *σ_UP_, σ_DOWN_* for the up and down cortical states, respectively). The remaining samples were considered as indeterminate state. As done for the membrane potential, we set the minimum state duration equal to 100 ms and the minimum inter-state interval equal to 50 ms. To assess the performance of the algorithm, we computed Receiver Operating Characteristic (ROC, Figure 1—figure supplement 1e) curves for both up and down state detection: for up states, true positives are those time instants defined as ‘up’ on the basis of both membrane potential and LFP, whereas false positives are time instants defined as ‘down’ in the membrane potential, but classified as ‘up’ from the LFP signal. The analogous definitions were applied for down state detection.

To evaluate whether the number of spikes fired from interneurons during down states was significantly higher than what could be predicted if all spikes came from misclassified time bins (i.e. in which down states were falsely detected from the LFP), we implemented the following test. We computed empirically from the ground truth data the probability that a time bin (20 ms resolution) is wrongly classified as down state for each given value of *S_comb_*. For each interneuron, the overall probability that all its putative down-state spikes were fired in misclassified bins was calculated taking the product over all spikes of down-state misclassification probability given the corresponding *S_comb_* value. Due to the rarity of down-state spikes, we assumed that the probability of misclassifying a bin containing a spike is roughly independent of that of other bins. This allowed us to compute the overall probability that all spikes come from misclassified bins as the product over all bins containing spikes of single-bin misclassification. This number thus quantifies the probability of the null hypothesis to be true (i.e. all interneurons’ spikes are fired in misclassified bins, hence interneurons never fire during down state), and was considered as p-value for each single interneuron.

All the analyses were performed by using custom-made software implemented in MATLAB (RRID:SCR_001622, The Mathworks, Natick, MA, USA).

### Phase locking and causal analysis between PV/SST interneurons and LFP

To investigate the temporal relationship between PV/SST interneurons’ spiking activity and up/down state occurrence during spontaneous activity, we asked whether the LFP slow oscillation phase (which in turn reflects cortical state) is related to the occurrence of spikes in PV/SST interneurons by quantifying the phase locking of spikes. To do that, we applied the method detailed in (Eschenko et al., 2012; Siapas et al., 2005). Briefly, we computed the instantaneous low-frequency phase of the LFP as the angle of the Hilbert transform of the LFP trace filtered in the [0.1, 4] Hz band. The phase of firing distribution quantifies, for each cell, the phase values at which each spike was fired. Non-uniform phase of firing distributions mean that neurons fire preferentially at certain phases. Hence, phase locking can be detected by assessing departure from uniformity in the distribution of rescaled phases observed at spike times. To correct for the effect of possible non-uniformities in the phase distributions due to asymmetries in the LFP wave shape (Siapas et al., 2005), we made the overall distribution of phase across all time points uniform by rescaling it by its cumulative distribution. The significance of phase locking was computed as departure from uniformity of the phase of firing distribution, using the Rayleigh’s test (Siapas et al., 2005). To infer possible causal dependencies between spikes and phase time series, we also computed how the phase locking between spike train and LFP (p<0.01, Bonferroni corrected) varies when the two signals are shifted in time with respect to the other. We computed the phase of firing distribution for each shift value τ of the LFP trace with respect to the spike train between −0.5 s and 0.5 s. For each of these distributions, we measured the strength of locking as one minus the circular variance, to quantify the concentration of the distribution of angles (Eschenko et al., 2012; Montemurro et al., 2008). To reliably estimate phase of firing distributions, we restricted the analysis to those cells firing at least 100 spikes in the whole recording (16/16 for PV cells, 15/19 for SST cells).

To estimate the temporal extent of the putative causation of interneurons on slow wave dynamics, we considered the range of negative time shifts τ for which the time-shifted phase locking was both statistically significant and, more conservatively, higher than the maximal value of time shifted locking over τ ≥ 0: indeed, no locking value at τ ≥ 0, by definition, can capture any causal relationships of spikes on slow waves (instead, locking values at τ ≥ 0 can be significant due to e.g. the autocorrelation of the phase time series and the entrainment of spike to slow oscillations). These putative causation time ranges have naturally the form τ_end_ ≤ τ < 0 (for two PV cells where τ_end_ was less than −0.5 s we conservatively set τ_end_ = −0.5 s). For each cell, we further determined the time shift corresponding to the maximum strength of locking (τ_*max*_).

We also determined the preferred phase of firing at τ = 0, τ = τ_*max*_ and τ = τ_end_, by calculating the circular median of the phase of firing distribution. Moreover, for each cell we computed the width of the phase of firing as its interquartile range. To estimate phase values associated to the start and the end of up/down states we considered the statistical distributions of instantaneous phase at state onset/offset detected from the LFP as reported in Figure 1—figure supplement 1h,i. We determined the start or the end of the state as the phase value associated to the peak of the distribution and we considered these values to determine the boundaries of up and down state displayed in Figure 2c,e,i,k. To better investigate the nature of the phase of firing distributions, we computed the phase of firing histograms for each neuron in that state, and separately for up, down and transition (down-to-up and up-to-down) states, in two different ways (Figure 2—figure supplements 1 and 2). The first quantification (phase of firing strength), that identifies the phase bins in which more spikes are fired, computed in each phase bin the number of spikes per phase bin averaged across all occurrences of a state. The second quantification (phase of firing reliability), that identifies the phase bins in which spikes are discharged more reliably across the occurrences of a state, computed in each phase bin the fraction of occurrences of a state in which we observed at least one spike. The two quantifications, for each phase bin, coincide if a neuron fires one spike in that phase bin. The two quantifications differ if a neuron fires sometimes more than one spike per phase bin and this number varies across different occurrences of a state. The similarity between phase of firing strength and phase of firing reliability (pooling up, down and transition states together) was evaluated, separately for each neuron, as the Pearson correlation coefficient across phase bins of the histograms of these two quantities. The population averaged Pearson correlation was: 0.986 ± 0.004 for N = 16 PV cells, and 0.996 ± 0.001 for N = 15 SST cells, p<0.05 for all correlations calculated in individual cells.

Finally, to assess the nature of the changes in the spike-triggered LFP over the putative causal time range, we looked for statistically detectable differences in the LFP phase speed (i.e. the rate of change of the LFP phase) across the spike times, over timescales of the order of τ_end_. More specifically, we considered, separately for up and down states and for PV and SST interneuron firing activity, all of the spikes occurring in a window between – X and – 200 ms before the end of state (where X was varied parametrically between – 400 and – 300 ms). We included only data up to – 200 ms before end of state because this was the range of causation exerted by the interneuron’s firing on phase dynamics that we found in real data (see Results). To test whether there was a difference between phase speed pre- and post-spike we computed, separately for each population of interneurons and for up and down states, the time-average of the phase speed over a time window T around the spike time (T varied between 50 and 200 ms). We then compared the pre- and post-spike results with a t test. We checked that the results of these comparisons were consistent across the different choices of the parameters X and T. Results of Figure 2 and Figure 2—figure supplement 3 were computed for X = - 400 ms and T = 200 ms. To check that the results of the statistical tests are not due to intrinsic asymmetries of the LFP dynamics close to state end, we repeated the same comparisons with control data. For each data stretch of phase speed around a spike time included in the analysis above, we selected a ‘control stretch’ of LFP phase speed as follows: we first computed the time average of the data stretch over the whole 400 ms interval centered on the corresponding interneuron spike time, then we randomly chose an equally long control stretch recorded when the interneuron was silent, whose distance from the respective state end was equal to the distance of the data stretch from its state end, and whose time average over the respective whole 400 ms interval differed from the data stretch time-average by less than 50 deg/s. We then triggered the phase speed change analysis to the centers of the control stretches rather than to the timings of a spike, which are the centers of the data stretches.

### Analysis of in vivo intracellular recordings

Recordings were inspected *a posteriori* to determine whether the optogenetic stimulus was delivered during an up or during a down state. A stimulus was delivered during an up state if the membrane potential of cell was stably depolarized (>10 mV) over the resting potential of that cell in a time window of 100 ms before the light stimulus. A stimulus was delivered during a down state if the membrane potential of cell was stably within ±3 mV from the resting potential of that cell in a time window of 100 ms before the light stimulus. Trials which did not satisfy either criterion were not considered for analysis. For photostimulation experiments in Figures 3 and 6 and Figure 6—figure supplement 4, the change in membrane voltage (ΔmV) was calculated in a time window before (Pre, duration, 100 ms and 50 ms for experiments in anesthetized or non-anesthetized animals, respectively) and after (Post, duration, 100 ms) light stimulation in 10 representative stimulation trials per cell. In photoinhibition experiments in which the membrane potential was measured (Figures 4 and 6 and Figure 6—figure supplement 4), three time windows were considered: Pre (duration, 100 ms and 50 ms for anesthetized or non-anesthetized experiments, respectively), Light (duration, 500 ms), and Post (duration, 100 ms). In photoinhibition experiments in which the spike frequency was evaluated, longer time windows were considered: Pre (duration, 1 s), Light (duration, 500 ms), and Post (duration, 1 s). In Figure 5, only the recordings in which stimulation occurred during a down state were considered. The onset of the optogenetically triggered up state was determined as the time at which the cell membrane potential crossed a threshold set at two times the standard deviation of the average down state membrane voltage value which was calculated in the Pre time window. The standard deviation associated to the onset was evaluated for each single neuron and its average across cells was used to estimate the temporal variation (jitter) for the triggered up state. To evaluate the membrane potential speed in pyramidal neurons during up-to-down (Figure 3—figure supplement 3) and down-to-up (Figure 5—figure supplement 1) state transitions, we performed a linear regression of the membrane potential as a function of time during the transition (Sanchez-Vives et al., 2010) and we considered its angular coefficient (i.e., the slope). To compare optogenetically-evoked transitions with spontaneously occurring transitions, for each recorded cell we manually selected ten evoked and ten spontaneous transitions and we evaluated the average slope of selected transitions across cells. To estimate the time lag between the up-to-down or the down-to-up transition in the experiments reported in Figure 6 (Figure 6—figure supplement 3), we calculated the onset of each transition as the time at which the cell membrane potential crossed a threshold set at two times the standard deviation of the average down, or up state membrane voltage value calculated in the Pre time window. The difference between the onset of transition from the cell recorded in channel two and the transition onset from the cell in channel one was considered as the transition time lag (Ch2-Ch1 delay in Figure 6—figure supplement 3).

### Analysis of in vivo extracellular recordings

LFP and MUA signals were analysed with custom made software programmed in MATLAB. Extracellular signals were inspected *a posteriori* to determine whether the optogenetic stimulus was delivered during an up or during a down state, similarly to what done for intracellular recordings. Up and down states were identified based on the presence of high frequency oscillations in the LFP or the appearance of extracellular spikes in the MUA. LFP traces were downsampled by a factor of 50 (from 50 kHz to 1 kHz) and low-pass filtered using a Chebyshev Type I filter (corner frequency, 100 Hz). The frequency content of the LFP was evaluated with spectrograms computed using 100 ms Hamming windows shifted every 1 ms. The one-sided modified periodogram estimate of the power spectral density (PSD) was computed (FFT length: 500 points) for each window and plotted as a function of time and frequency (0–100 Hz) in a logarithmic scale. A blanking window of 10 ms at the beginning of the Pre and Post windows was used to exclude stimulation artefacts. The relative power in the low gamma (30–60 Hz) and high gamma (60–90 Hz) range was computed by integrating the PSD function over the two frequency ranges and normalizing the integral over the total signal power in the pre-stimulus window. The PSD is estimated by means of the Welch’s averaged modified periodogram spectral estimation method (segment length: half of the considered window; overlap: 50%) (Welch, 1967).

The MUA signal was isolated by high-pass filtering (corner frequency, 300 Hz). Spikes were identified based on a threshold set at five times the standard deviation of the noise. 100 ms time binning (10 ms for the dual MUA recordings) was used to compute spike count histograms. Spike frequency was obtained as the total number of identified spikes in the different windows (Pre, Light, and Post) over the temporal duration of the window. The MATLAB source code used to perform these calculations is available as a supplementary material file (Source code 2).

### Study design and statistics

For each experimental group, sample size was chosen based on previous in vivo studies (Beltramo et al., 2013). No statistical methods were used to predetermine sample size. All recordings with no technical issues were included in the analysis. Values are expressed as mean ± s.e.m, unless otherwise stated. The Kolmogorov-Smirnov test was run on each experimental sample to test for normality. Two-tailed (unless otherwise stated) Student’s *t*-test (in case of normal distribution), and the Mann-Whitney or Wilcoxon signed-rank (for unpaired and paired comparison of non-normal distributed data, respectively) tests were used when comparing two populations. For comparison of more than two populations, one-way ANOVA with Bonferroni *post-hoc* test was used for normally distributed data; otherwise, the non-parametric Friedman with Dunn *post-hoc* test was applied. Statistical analysis was performed using OriginLAB (RRID:SCR_002815), GraphPad Prism (RRID:SCR_002798), or MATLAB (RRID:SCR_001622) software.

## Additional files

Source code 1. UP/DOWN state detection on LFP recordings and quantification of inhibitory interneurons firing properties. Functions and scripts contained in this file have been used to produce data and plots reported in Figure 1 and Figure 1—figure supplement 1. **DOI:** http://dx.doi.org/10.7554/eLife.26177.059

Source code 2. Analysis of LFP and MUA recordings during optogenetic manipulation of interneurons. Functions and scripts contained in this file have been used to produce data and plots reported in Figure 3—figure supplements 4 and 5 and Figure 4—figure supplements 3 and 4. **DOI:** http://dx.doi.org/10.7554/eLife.26177.060
