# Supplementary material for: An inhibitory gate for state transition in cortex
Source: eLife. 2017 May 16;6:e26177. doi: 10.7554/eLife.26177 (PMC5444901; doi:10.7554/eLife.26177)
Supplement: Related texts

# Article commentary: Decision letter

**DOI:** 10.7554/eLife.26177.061

**Affiliations:** Stanford University School of Medicine, United States

In the interests of transparency, eLife includes the editorial decision letter and accompanying author responses. A lightly edited version of the letter sent to the authors after peer review is shown, indicating the most substantive concerns; minor comments are not usually included.

Thank you for submitting your article "An inhibitory gate for state transition in cortex" for consideration by *eLife*. Your article has been reviewed by two peer reviewers, and the evaluation has been overseen by a Reviewing Editor and Andrew King as the Senior Editor. The following individuals involved in review of your submission have agreed to reveal their identity: Michael M Halassa (Reviewer #1); Antoine Adamantidis (Reviewer #2).

The reviewers have discussed the reviews with one another and the Reviewing Editor has drafted this decision to help you prepare a revised submission.

Summary:

This study provides important data and quantitative analysis of the roles of parvalbumin (PV) and somatostatin (SST) inhibitory interneurons in controlling sleep-related cortical up-to-down and down-to-up state transitions using in vivo patch-clamp recording/imaging and optogenetics approaches in both anesthetized and non-anesthetized mice. Both PV and SOM cells were found to be active during the UP states and phase locked to the local field potential slow wave oscillation and are causally involved in the termination of the up state. This was shown using electrophysiological recording during both spontaneous bistable state transitions and with optogenetic activation of PV/SST neurons during up states.

Silencing of these cells during up states prolonged up states, but Interestingly their silencing during down states revealed different dynamics. Silencing of PV cells led to a faster and higher pyramidal cell firing during up states than silencing of SST cells, suggesting alternative mechanisms.

The results are clearly discussed in light of recent studies, in particular regarding the role of thalamus versus cortical circuits in up/down state transitions.

Essential revisions:

Please provide additional analyses to address the following reviewers' concerns:

Analysis:

1) There is great appeal in the fact that this is not an optogenetic-based study, and that the authors spent considerable effort characterizing the basic relationship between PV/SST spike times and LFP phase. However, there could be some more things to interrogate in this dataset that would enhance overall clarity:a) The individual phase histograms of each neuron within the PV and SST datasets could use a little more characterization. How much variability is associated with each bin? That helps clarify how consistent a neuron fires with respect to a specific slow oscillation phase. This would be a starting point to figuring out whether there are phases of consistent spiking (don't have to be the dominant ones) associated with UP to Down state transitions in some neurons; imagine that a subset of PV and/or SST neurons shows consistent spiking at a specific phase close to the UP to Down state transition. Taking a median statistic for each distribution will miss that, and would miss important potential differences between PV and SST neurons. Plus, simply counting spikes per bin would not get at that because a bin containing 10 spikes could have gotten those in one slow oscillation cycle or consistently on each of the 10 slow oscillation cycles that were recorded. These two scenarios would have very different interpretations. It may ultimately end up being the case that only a subset of these broad interneuronal types are engaged in regulating this transition, so the overall theoretical framework guiding these experiments (a discussion point) would benefit from approaching the data analysis that way. In relation to this point, and along the lines of examining whether certain neurons preferentially participate in the aforementioned transitions, the authors could look to see whether any of the phase distribution histogram features correlates with speed of transition on a neuron-by-neuron basis.b) Regardless of the point above, the phase speed analysis is quite informative, and it would be highly desirable to see comparable analysis done on the optogenetic manipulation experiments too. It's sort of clear, by eye, that the dV/dt in Figure 3 is larger for SST than PV manipulation. Doing this analysis may help to clarify which of those two cell types (broadly defined) contain populations that are more likely to be the natural source for UP to Down state transition. That will make the point substantially stronger.c) The same is true for Figure 4; the slope of the Up to Down State transition appear to be different under the two Arch manipulations. The associated analysis misses this point a little, but compare the membrane potential changes with and without light when it's clear that disinhibition will cause some level of depolarization. The important point to clarify here is whether the transition slopes change in a systematic way which can be compared to the baseline phenomena in Figure 2.

2) The authors state that locally controlled up/down states in the cortex rapidly spread over mesoscale cortical areas, leading to a "near synchronicity" of up/down state during both optogenetic activation and silencing of (LII/III) PV and SST cells. While this is very interesting, the manuscript would greatly benefit from some additional analysis of this synchronicity. From Figure 6 it is difficult to assess the time lags of Up/down states recording 2mm apart in the cortex.

# Reply: Author response

**DOI:** 10.7554/eLife.26177.062

*Essential revisions:*

*Please provide additional analyses to address the following reviewers' concerns:*

*Analysis:*

*1) There is great appeal in the fact that this is not an optogenetic-based study, and that the authors spent considerable effort characterizing the basic relationship between PV/SST spike times and LFP phase.*

We thank the referee for his/her comment. The appeal of combining optogenetics with careful analysis of unperturbed neural activity was highlighted in the Discussion of the previous version of manuscript and is further emphasized in the new version (”Interneurons control the up-to-down transition”).

However, there could be some more things to interrogate in this dataset that would enhance overall clarity:a) The individual phase histograms of each neuron within the PV and SST datasets could use a little more characterization. How much variability is associated with each bin? That helps clarify how consistent a neuron fires with respect to a specific slow oscillation phase. This would be a starting point to figuring out whether there are phases of consistent spiking (don't have to be the dominant ones) associated with UP to Down state transitions in some neurons; imagine that a subset of PV and/or SST neurons shows consistent spiking at a specific phase close to the UP to Down state transition. Taking a median statistic for each distribution will miss that, and would miss important potential differences between PV and SST neurons. Plus, simply counting spikes per bin would not get at that because a bin containing 10 spikes could have gotten those in one slow oscillation cycle or consistently on each of the 10 slow oscillation cycles that were recorded. These two scenarios would have very different interpretations. It may ultimately end up being the case that only a subset of these broad interneuronal types are engaged in regulating this transition, so the overall theoretical framework guiding these experiments (a discussion point) would benefit from approaching the data analysis that way.

*T*o investigate whether there was a possible dissociation between phases with highest average firing rate and phases with most consistent spiking, we compared the phase of firing strength, which identifies the phase bins in which more spikes are fired, with the phase of firing reliability, which identifies the phase bins in which spikes are discharged more reliably (Figure 2—figure supplement 1 and 2). The comparison was done for each cell and separately for up states, down states, state onsets and state offsets. The phase of firing strength was computed as the average number of spikes per phase bin in a single occurrence of a state. The phase of firing reliability instead computed in each phase bin the fraction of states in which at least one spike was observed. For each phase bin, the two quantities coincide if a neuron always fires a single spike in that phase bin across occurrences of state. In contrast, the two quantities differ if a neuron fires unreliably in that phase bin across trials. We found that the histograms of the phase of firing strength and of the phase of firing reliability were highly correlated for all recorded cells (average Pearson correlation: 0.986 ± 0.004 for N = 16 PV cells, and 0.996 ± 0.001 for N = 15 SST cells, p < 0.05 for all correlations calculated in individual cells). This was due to the fact that the vast majority (96.1 ± 0.8% for N = 16 PV interneurons, 99.5 ± 0.4% for N = 15 SST cells) of phase bins (bin width: 10 ms) contained only either zero or one spike. Overall, this additional analysis indicates that the phase bins characterized by stronger firing are also those in which individual neurons fire more reliably.

*In relation to this point, and along the lines of examining whether certain neurons preferentially participate in the aforementioned transitions, the authors could look to see whether any of the phase distribution histogram features correlates with speed of transition on a neuron-by-neuron basis.*

To address the referee’s request, we first computed the spike-triggered phase speed change for each individual neuron (Figure 2 displays the phase speed changes pooled across all neurons of each class). In doing so, we focused only on phase speed changes triggered by spikes in the up state, because only in this case enough spikes per neuron for a robust single-cell phase-speed-change analysis were observed. We then analyzed the correlation of the phase speed change of each cell with several different features of the phase of firing distribution.

We found significant correlation between the single-cell phase speed change and the circular variance of the phase of firing distribution, which quantifies the width of the distribution, for both PV (correlation 0.67, p = 0.005, N = 16) and SST interneurons (correlation 0.58, p = 0.03, N = 14). Cells with particularly large distributions (circular variance ≥ 0.35) tended to show large speed change (Figure 2—figure supplement 4a), suggesting that cells whose firing covers larger portions of the up state may have a larger causal effect.

We also found correlation between single-cell phase speed changes and the preferred phase of firing for both PV (circular-linear correlation 0.60, p = 0.05, N = 16) and SST (circular-linear correlation 0.74, p = 0.02, N = 14). Cells with earlier preferred phase of firing tended to show large speed change (Figure 2—figure supplement 4b). Preferred phase of firing and circular variance however tended to be correlated across cells (Figure 2—figure supplement 4c), making it difficult to dissociate the independent effect of each of these two variables on phase speed changes. These data suggest that specific features of the phase of firing distribution may be used as indicators of the causal role of interneurons in the slow wave cycle, and we plan to investigate closely this matter in future dedicated experiments.

*b) Regardless of the point above, the phase speed analysis is quite informative, and it would be highly desirable to see comparable analysis done on the optogenetic manipulation experiments too. It's sort of clear, by eye, that the dV/dt in Figure 3 is larger for SST than PV manipulation. Doing this analysis may help to clarify which of those two cell types (broadly defined) contain populations that are more likely to be the natural source for UP to Down state transition. That will make the point substantially stronger.*

As requested by the referee, we quantified the slope of the up-to-down state transitions in individual cells for the experiments displayed in Figure 3. We found that optogenetic activation of PV and SST interneurons during ongoing up states triggered up-to-down state transitions with larger slope compared to spontaneous up-to-down transitions observed at the level of the membrane potential in the same intracellularly recorded cells. The slope of up-to-down transition was not significantly different for optogenetic stimulation of PV and for optogenetic stimulation of SST interneurons. These data are now reported in subsection *“*Optogenetic activation of interneurons triggers up-to-down transitions” and in Figure 3—figure supplement 3. As a consequence of these new results, we also modified Figure 3 to show more representative traces that display similar transition slope following stimulation of PV and SST interneurons.

*c) The same is true for Figure 4; the slope of the Up to Down State transition appear to be different under the two Arch manipulations. The associated analysis misses this point a little, but compare the membrane potential changes with and without light when it's clear that disinhibition will cause some level of depolarization. The important point to clarify here is whether the transition slopes change in a systematic way which can be compared to the baseline phenomena in Figure 2.*

We performed the additional analysis requested by the reviewer and quantified the slope of the optogenetically-induced transitions in Figure 4. We found that optical inhibition of PV cells triggered down-to-up state transitions with larger slope compared to spontaneous events. In contrast, optical suppression of SST interneurons elicited down-to-up transitions with slope values that were undistinguishable from those of spontaneous down-to-up transitions. To compare between optogenetically-induced and spontaneous transitions we used an internal control, i.e. spontaneous down-to-up transitions measured at the level of the membrane potential in the same cells in which the optogenetically-induced transitions were recorded. We believe this is the appropriate control as it compares the slope of state transitions (either spontaneous or optogenetically-induced) measured with the same technique (intracellular recordings) and in the same set of cells, rather than comparing with data in Figure 2 where state transitions were measured in a different set of experiments with the LFP. This additional analysis is now reported in subsection “Inhibitory control of state transition is interneuron subtype-specific” and in Figure 5—figure supplement 1.

*2) The authors state that locally controlled up/down states in the cortex rapidly spread over mesoscale cortical areas, leading to a "near synchronicity" of up/down state during both optogenetic activation and silencing of (LII/III) PV and SST cells. While this is very interesting, the manuscript would greatly benefit from some additional analysis of this synchronicity. From Figure 6 it is difficult to assess the time lags of Up/down states recording 2mm apart in the cortex.*

The distributions of the time lag of up-to-down and down-to-up transitions and their mean values are now reported in subsection “Local optogenetic manipulation of interneurons induces mesoscale state transitions” and in Figure 6—figure supplement 3.
